# Disruption of cysteine metabolism leads to synthetic lethality and *in vivo* fitness impairment in *Acinetobacter baumannii*

**DOI:** 10.1128/mbio.00842-26

**Published:** 2026-06-15

**Authors:** Avik Pathak, Snehlata Saini, Ranjana Pathania

**Affiliations:** 1 Department of Biosciences and Bioengineering, Indian Institute of Technology Roorkee 30112, Roorkee, Uttarakhand, India; 2 Centre of Excellence in Disaster Mitigation and Management, Indian Institute of Technology Roorkee 30112, Roorkee, Uttarakhand, India; Universiteit Gent, Gent, Belgium

**Keywords:** bacterial metabolism, reactive oxygen species (ROS), antibiotics, bacterial pathogenesis, bioenergetics

## Abstract

*Acinetobacter baumannii* is a critical nosocomial pathogen that causes infections in diverse host niches, including the respiratory tract, bloodstream, and urinary tract. Considering the contemporary landscape of antibiotic resistance, alongside targeting resistance determinants, there is a need to identify novel and alternative targets for therapeutic intervention. Central metabolism plays a major role in the success of a pathogen and represents a pool of plausible drug targets. However, metabolic networks are inherently complex and often employ redundant pathways to ensure the supply of essential metabolites. In our efforts to delineate the metabolic pathways contributing to the pathophysiological fitness of *A. baumannii*, we focused on biosynthesis and uptake mechanisms that maintain intracellular cysteine homeostasis. Cysteine is one of the least abundant amino acids in proteins; however, it is crucial for the folding and catalytic activity of enzymes involved in diverse pathophysiological pathways. We show that *A. baumannii* possesses two partially redundant serine acetyltransferases involved in cysteine biosynthesis. Disruption of cysteine biosynthesis affects the intracellular cysteine pool, leading to metabolic dysregulation. The metabolic dysregulation further leads to a defect in cellular bioenergetics, oxidative stress mitigation, and antibiotic stress survival *in vitro* and *in vivo*. Combinatorial disruption of cysteine biosynthesis and cystine transport results in synthetic lethality and fitness attenuation within the host. Our work provides insights into the metabolic complexities and vulnerabilities of *A. baumannii* and underscores the therapeutic potential of targeting pathways that sustain cysteine homeostasis, including combination strategies, to combat infections caused by this priority pathogen.

**IMPORTANCE:**

*Acinetobacter baumannii* is a critical nosocomial pathogen, and the rising prevalence of antibiotic resistance in this organism necessitates the identification of novel therapeutic targets. Central metabolism, particularly amino acid metabolic pathways, represents a promising yet underexplored area for such interventions. Cysteine, a sulfur-containing amino acid, is essential for the activity of different enzymes involved in key physiological processes. In this study, we demonstrate that the cysteine biosynthesis pathway plays a critical role in maintaining metabolic homeostasis and survival under antibiotic stress while exhibiting functional redundancy with the cystine uptake system in sustaining *in vivo* fitness in *A. baumannii* . Simultaneous disruption of cysteine biosynthesis and uptake results in synthetic lethality and a marked fitness defect in a murine pneumonia model. Our work reveals metabolic complexities and highlights the metabolic vulnerabilities of *A. baumannii*, which can be further explored for therapeutic interventions to curb infections caused by this priority pathogen.

## INTRODUCTION

*A. baumannii* is a gram-negative coccobacillus and a nosocomial pathogen responsible for a wide spectrum of infections, including bloodstream infections, ventilator-associated pneumonia, urinary tract infections, meningitis, sepsis, and wound infections (1). The success of this pathogen can be attributed to its robust and adaptable metabolic network that enables survival across diverse host niches. The genome plasticity and exceptional ability to acquire antibiotic resistance genes have further led to an increase in resistance to several clinically relevant antibiotics, thereby limiting treatment options (1, 2). Inside the host, pathogens encounter a range of hostile conditions, including oxidative stress, nutritional immunity, and restricted availability of essential nutrients (3, 4). To persist and establish infection, pathogens need to modulate their metabolism to ensure the supply of essential nutrients, including energy sources and metal ions. Research focused on *A. baumannii* metabolism, especially amino acid metabolism, has highlighted pathways critical for survival and for competing with the resident microbiota inside the host. *A. baumannii* is a major cause of ventilator-associated pneumonia, and the human lungs contain diverse amino acids available to *A. baumannii* as carbon and nitrogen sources. Recent studies have highlighted the importance of amino acid catabolic pathways in colonization of the pulmonary niche by *A. baumannii*. HutH, involved in histidine catabolism and the arginine succinyltransferase (Ast) pathway, which is involved in arginine catabolism, has been shown to play a critical role in the establishment of pneumonia by *A. baumannii* (5, 6). Beyond the lungs, amino acid catabolism has also been shown to influence *A. baumannii* gut colonization, where *A. baumannii* utilizes ornithine, a non-preferred carbon source, to compete with the resident microbiota (7). Furthermore, to overcome host-mediated nutritional immunity, *A. baumannii* employs specialized transporters to take up essential metal ions (8). These studies highlight the complexities of metabolic circuits in *A. baumannii* and their importance in colonization and establishment of infection in the host. Considering the contemporary antibiotic resistance landscape, there is a dire need to identify non-conventional pathways for therapeutic interventions, and such metabolic pathways, central to the *in vivo* fitness of *A. baumannii*, represent a pool of plausible drug targets.

In this study, we focus on cysteine, a sulfur-containing amino acid that is crucial for the function of several enzymes, including those involved in peptidoglycan biosynthesis, peptidoglycan recycling, cofactor biosynthesis, disulfide linkage formation in periplasmic proteins, and the mitigation of oxidative stress (9–12). Cysteine is also a precursor of glutathione, a compound involved in oxidative stress mitigation (9). The cysteine biosynthesis pathway in bacteria differs from that in mammals, making it an attractive target for therapeutic interventions (13, 14). However, this requires a systematic investigation, as pathogens often possess alternative pathways to sustain intracellular levels of critical metabolites (15, 16).

In most bacteria, cysteine biosynthesis proceeds via a two-step process. First, a single serine acetyltransferase catalyzes the formation of O-acetylserine from L-serine and acetyl-CoA. Subsequently, O-acetylserine is converted to cysteine by two cysteine synthases, with H₂S serving as the sulfur donor (17). In addition to *de novo* synthesis, some pathogens can also acquire cystine, the oxidized form of cysteine, which is converted to cysteine (18). In organisms such as *Brucella ovis*, cysteine biosynthesis is important for growth and fitness in intracellular niches (19). In *Mycobacterium tuberculosis*, the serine acetyltransferase CysE has been shown to be important for oxidative stress management and *in vivo* fitness (20). In *A. baumannii*, transcriptional regulators Cbl and GigC have been shown to be involved in sulfate assimilation, among other regulatory roles, and are important for fitness in *Galleria mellonella* and murine pneumonia infection models, respectively (21, 22).

To date, the *de novo* cysteine biosynthesis pathway and the uptake dynamics sustaining intracellular cysteine homeostasis and its importance in the pathophysiology of *A. baumannii* have not been studied. Through enzymatic assays and mutation studies, we show that, in *A. baumannii*, the first step in cysteine biosynthesis from L-serine is catalyzed by two partially redundant serine acetyltransferases, CysE and SAT. Deletion of both of these genes abolishes growth in cysteine-deficient medium, confirming this as the sole biosynthetic route. The mutant can grow in LB broth, a complex medium, due to the presence of a cystine transporter; however, cystine uptake fails to maintain intracellular cysteine levels. Depletion of the intracellular cysteine pool leads to widespread metabolic dysregulation, affecting the levels of critical metabolites, including peptidoglycan precursors, vitamins and cofactors, and antioxidant molecules. Perturbations in metabolic homeostasis further result in increased sensitivity to antibiotics *in vitro* and *in vivo*. Combinatorial deletion of biosynthesis and uptake genes results in synthetic lethality, a phenomenon in which the combination of genetic perturbations produces a more profound effect (23) and *in vivo* fitness attenuation. Collectively, our findings reveal key metabolic vulnerabilities in *A. baumannii* and highlight the therapeutic potential of targeting cysteine homeostasis to mitigate infections caused by this pathogen.

## RESULTS

### In the *A. baumannii genome*, two genes annotated as serine o-acetyltransferase and serine acetyltransferase encode functional enzymes

**Fig 1:**
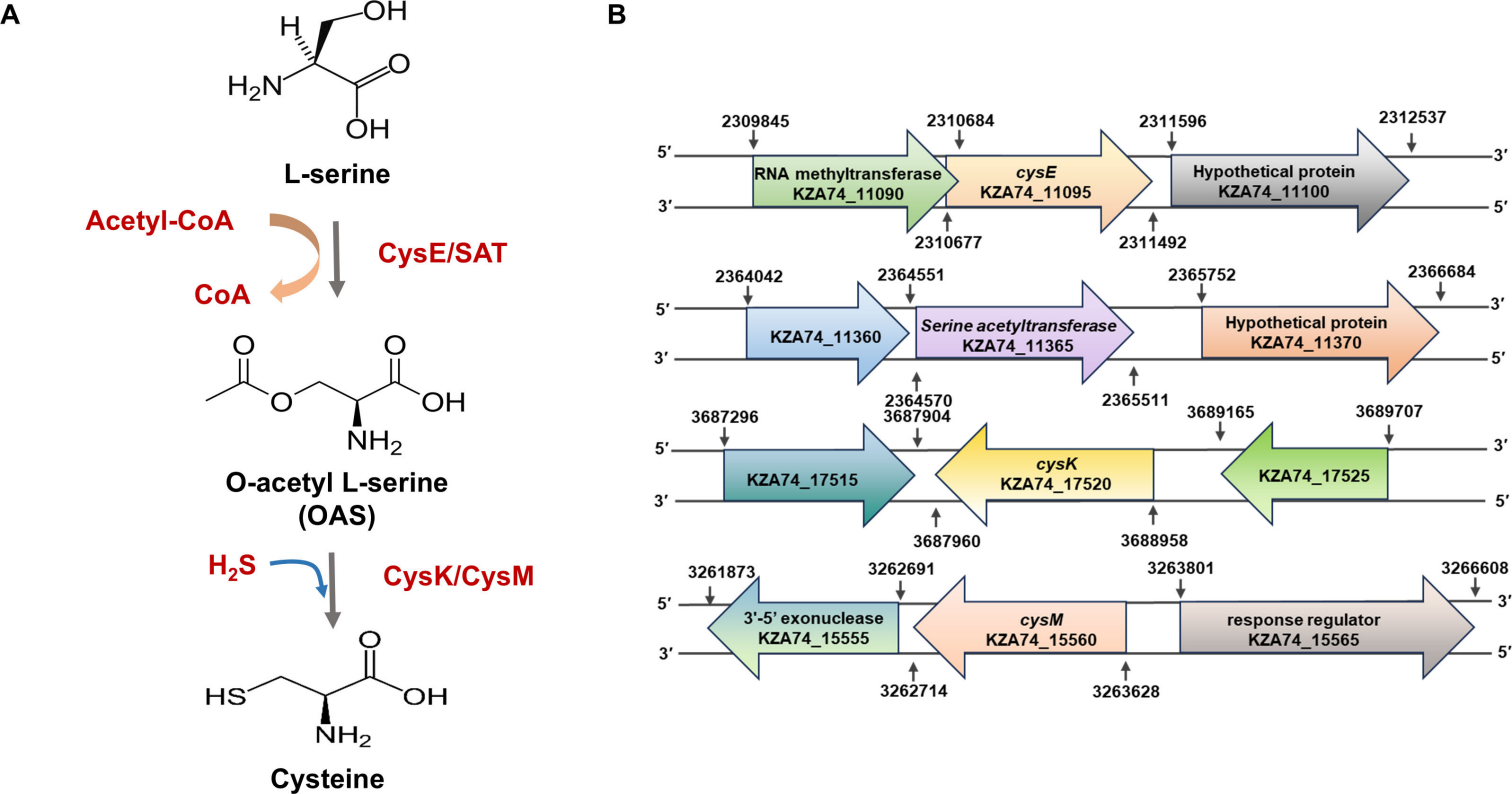
Cysteine biosynthesis pathway in *Acinetobacter baumannii.* (**A**) Cysteine is synthesized from L-serine and acetyl-CoA. The first step is catalyzed by two serine acetyltransferases, CysE and SAT, which transfer the acetyl group to L-serine, thereby synthesizing O-acetyl-L-serine. In the second step, a sulfur group is transferred to O-acetyl-L-serine from H_2_S by two cysteine synthases, CysK and CysM, to synthesize cysteine. (**B**) Genomic organization of *cysE*, *sat*, *cysK,* and *cysM*.

In general, prokaryotes possess a single serine acetyltransferase, whereas plants have multiple isoforms localized in distinct cellular compartments. These isoforms vary in their activity and feedback regulation to cater to the differential needs of cysteine in different compartments within the cell (17, 24, 25). Interestingly, in *A. baumannii* ATCC17978 UN genome (NCBI reference sequence CP079931.1), there are two genes annotated as serine O-acetyltransferase (KZA74_11095) and serine acetyltransferase (KZA74_11365). In this study, we have referred to serine o-acetyltransferase as *cysE* and the serine acetyltransferase as *sat* (Fig. 1A and B). We also assessed their similarity to other bacterial serine acetyltransferases, including both gram-negative and gram-positive bacteria. CysE showed the highest similarity (62.57%) with *Helicobacter pylori* serine acetyltransferase (Fig. S1A and B), and SAT showed the highest similarity (46.29%) with *Bacillus subtilis* serine acetyltransferase (Fig. S2A and B), among the tested bacterial species. To assess whether both genes encode functional enzymes, we incubated the purified enzymes with acetyl-CoA and L-serine and monitored the breakdown of acetyl-CoA and the decrease in absorbance at 232 nm over time (26). We found that both purified enzymes could break acetyl-CoA, resulting in a decrease in absorbance at 232 nm, indicating that both annotated genes encode functional enzymes (Fig. S3A through D).

### *A. baumannii* possesses two functional cysteine synthases involved in the conversion of O-acetylserine to cysteine

There are two genes annotated as cysteine synthase in the *A. baumannii* genome, that is, cysteine synthase A or *cysK* (KZA74_17520) and cysteine synthase B or *cysM* (KZA74_15560) (Fig. 1A and B). To investigate their involvement in cysteine biosynthesis, we incubated purified enzymes with O-acetyl serine and Na_2_S and detected cysteine production using the ninhydrin method. Both enzymes were able to synthesize cysteine, confirming that both genes encode functional enzymes (Fig. S3E and F).

### Deletion of *cysE* and not *sat* impairs growth in a cysteine-deficient medium, while the double mutant fails to grow

**Fig 2:**
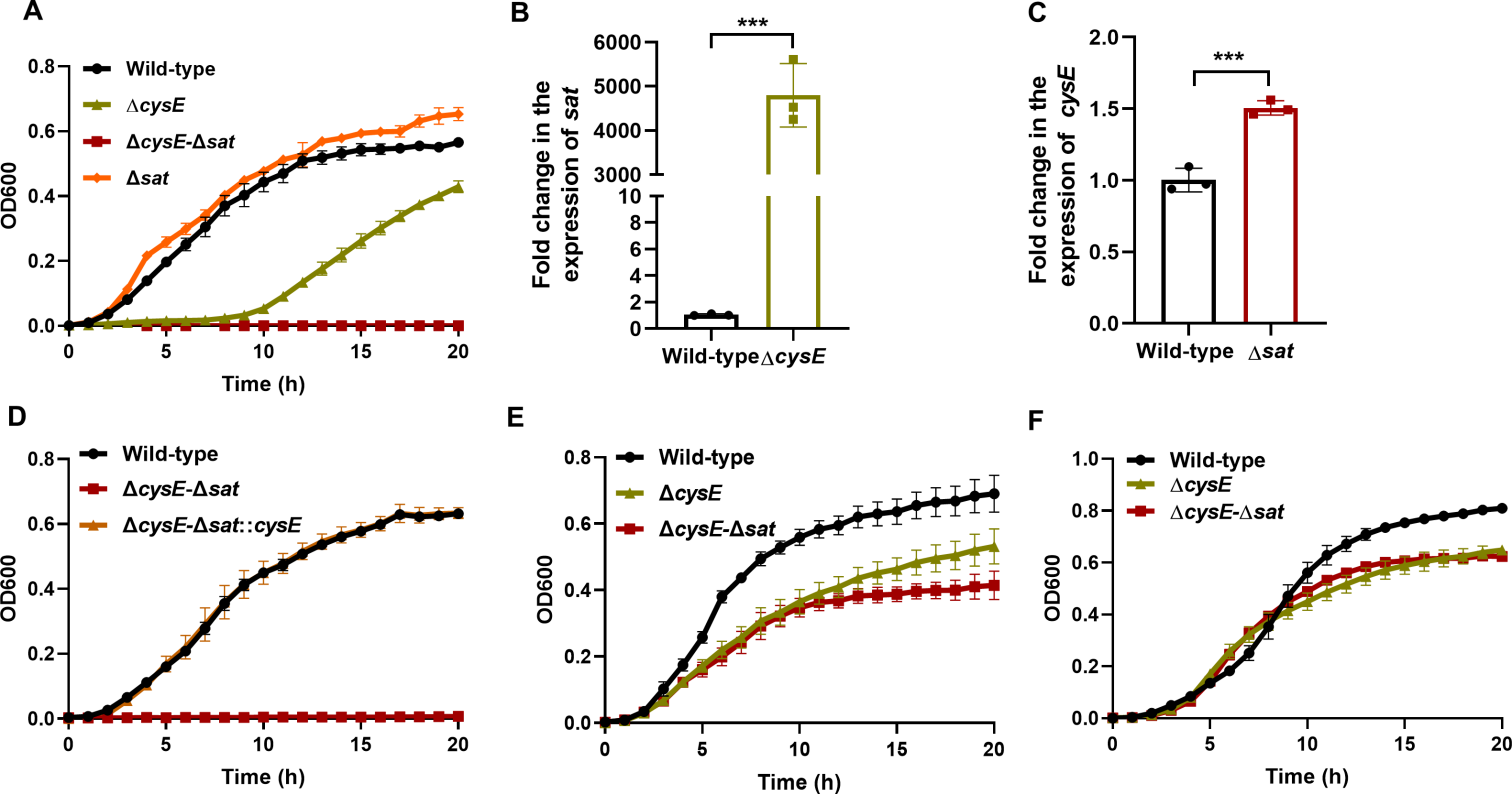
Deletion of *cysE* and not *sat* impairs growth in a cysteine-deficient medium, while the double mutant fails to grow. (**A**) Growth profile analysis of the wild-type, Δ*cysE*, Δ*sat,* and Δ*cysE-*Δ*sat* strains in M9-succinate medium. Each point represents the mean of three values with SD shown as error bars. (**B**) RT-PCR data depicting changes in the expression of *sat* in the Δ*cysE* strain as compared to the wild-type strain. The data represent values from three biological replicates with SD shown as error bars. Statistical significance was measured using a t-test, where *** represents *P*-value < 0.001. (**C**) RT-PCR data depicting changes in the expression of *cysE* in the Δ*sat* strain as compared to the wild-type strain. The data represent values from three biological replicates, with SD shown as error bars. Statistical significance was measured using a t-test, where *** represents *P*-value < 0.001. (**D**) Growth profile analysis of wild-type, Δ*cysE-*Δ*sat,* and Δ*cysE-*Δ*sat::cysE* strains in M9-succinate medium. (**E**) Growth profile analysis of the wild-type, Δ*cysE*, and Δ*cysE-*Δ*sat* strains in M9-succinate medium supplemented with 200 µM L-cysteine. (**F**) Growth profile analysis of the wild-type, Δ*cysE*, and Δ*cysE-*Δ*sat* strains in M9-succinate medium supplemented with 200 µM L-cystine. Each point represents the mean of three values with SD shown as error bars.

To study the effect of deletion of the cysteine biosynthesis genes on the pathophysiology of *A. baumannii*, we created deletion mutants of *cysE* (annotated as Δ*cysE*), *sat* (annotated as Δ*sat*), and a double deletion mutant of *cysE* and *sat* (annotated as Δ*cysE-*Δ*sat*) using a homologous recombineering approach (27, 28) and assessed their growth profiles in a cysteine-deficient medium, that is, M9 minimal salts supplemented with succinate as the sole carbon source, mentioned as M9-succinate hereafter. The Δ*cysE* strain showed a prominent growth defect, whereas the Δ*sat* strain showed no growth defect, and Δ*cysE*-Δ*sat* failed to grow (Fig. 2A). Although the Δ*cysE* strain exhibited a significant growth defect, it achieved substantial growth. By RT-PCR analysis, we found *sat* to be upregulated more than 1,000-fold in the Δ*cysE* strain compared to wild type (Fig. 2B); however, its contribution was insufficient to fully compensate for the loss of *cysE*, demonstrating partial redundancy. In enzymatic assays, we found that SAT activity was lower than that of CysE, which, to some extent, explains why upregulation of *sat* could not compensate for the loss of *cysE* (Fig. S3A through D). Interestingly, in the Δ*sat* strain, there was a modest upregulation of *cysE*, indicating differences in their transcriptional regulation (Fig. 2C). Genetic complementation with a functional *cysE* allele restored the growth of Δ*cysE-*Δ*sat* to wild-type level in M9-succinate medium (Fig. 2D). Supplementation of M9-succinate medium with cysteine (Fig. 2E) or cystine (Fig. 2F) could alleviate the growth defect associated with the cysteine biosynthesis mutants, indicating that *A. baumannii* possesses functional uptake systems. These observations also confirmed that the CysE- and SAT-mediated pathway is the sole cysteine biosynthesis pathway in *A. baumannii*.

### Disruption of cysteine biosynthesis alters cellular morphology but does not impair growth in complex medium

**Fig 3:**
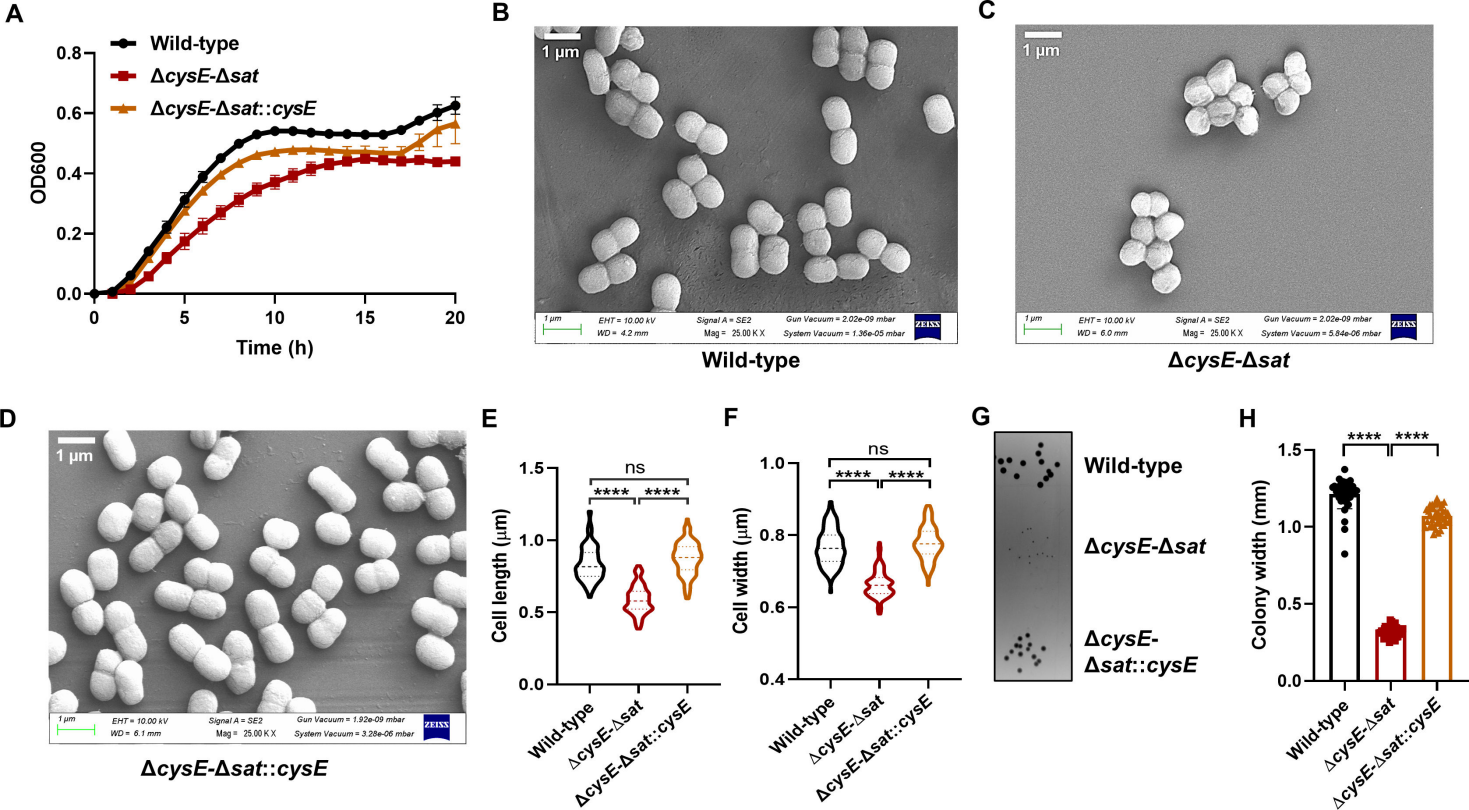
Cysteine auxotroph Δ*cysE*-Δ*sat* grows well in a complex medium but exhibits alteration in cellular morphology and colony size. (**A**) Growth profile analysis of wild-type, Δ*cysE*-Δ*sat*, and Δ*cysE*-Δ*sat::cysE* in LB broth. Each point represents the mean of three values, with the SD shown as error bars. Scanning electron micrographs of (**B**) wild-type, (**C**) Δ*cysE-*Δ*sat*, and (**D**) Δ*cysE-*Δ*sat::cysE* cells from logarithmic growth phase visualized at 25,000×. Cell length (**E**) and width (**F**) were quantified using ImageJ with reference to the scale and plotted using GraphPad Prism (*n* = 100 cells). Statistical significance was measured using one-way ANOVA with multiple comparisons, where **** represents *P*-value < 0.0001, and ns represents non-significant. (**G**) Image of colonies formed by wild-type, Δ*cysE-*Δ*sat,* and Δ*cysE-*Δ*sat::cysE* strains on an LB agar plate. Strains were grown till mid-log phase, diluted, and spotted onto LB agar plates, and incubated for 22 hours. (**H**) Distribution of colony widths measured using ImageJ and plotted using GraphPad Prism (*n* = 40 colonies). Statistical significance was measured using one-way ANOVA with multiple comparisons, where **** represents *P*-value < 0.0001.

When grown in Luria-Bertani (LB) broth, a complex medium, the cysteine biosynthesis mutants grew well, indicating that cystine uptake from the medium supports growth. In LB, cysteine is present as the oxidized form, cystine, which is the predominant form in an oxidized environment (29). Interestingly, there was a difference in OD_600_ between the wild type and the cysteine biosynthesis mutants, which was not replicated in a difference in colony-forming units (CFU) at endpoint (Fig. S4A and B). To further check for growth defects in terms of viable cell count and growth rate, we measured OD_600_ and CFU at the indicated time points and calculated the growth rate from CFU in the logarithmic phase of growth. Although the mutants exhibited lower OD_600_ values than wild type at the indicated time points, CFU counts were comparable, and there was no significant difference in growth rate (Fig. S4C through E). This led us to hypothesize that the disruption of cysteine biosynthesis might have resulted in an alteration in cellular morphology without affecting the growth rate, which, in turn, led to a change in the OD_600_ value. Indeed, through scanning electron microscopy of log-phase cells, we found that the Δ*cysE* strain exhibits a significant reduction in both cell length and width compared to the wild-type strain, with the Δ*cysE-*Δ*sat* strain displaying an even more pronounced effect (Fig. S5A through S5E). Differences in OD_600_ and defects in cell length and width observed in the Δ*cysE-*Δ*sat* strain during growth in LB broth were rescued by genetic complementation, confirming the role of cysteine biosynthesis in maintaining cellular morphology (Fig. 3A through F). In addition, we observed that when grown on an LB agar plate, the Δ*cysE* strain forms smaller colonies than the wild type, with an even greater reduction observed in the Δ*cysE-*Δ*sat* strain (Fig. S5F and G). The alteration in colony size upon disruption of cysteine biosynthesis has also been reported in *Rhodobacter capsulatus* (30). This defect was alleviated upon complementation of Δ*cysE-*Δ*sat* with *cysE* (Fig. 3G and H). We also found *that sat was* upregulated in Δ*cysE* compared to wild type when grown in LB broth (Fig. S5H). This indicates that *A. baumannii* also focuses on cysteine biosynthesis even when growing in complex medium containing cystine.

### Disruption of cysteine biosynthesis leads to metabolic perturbation

**Fig 4:**
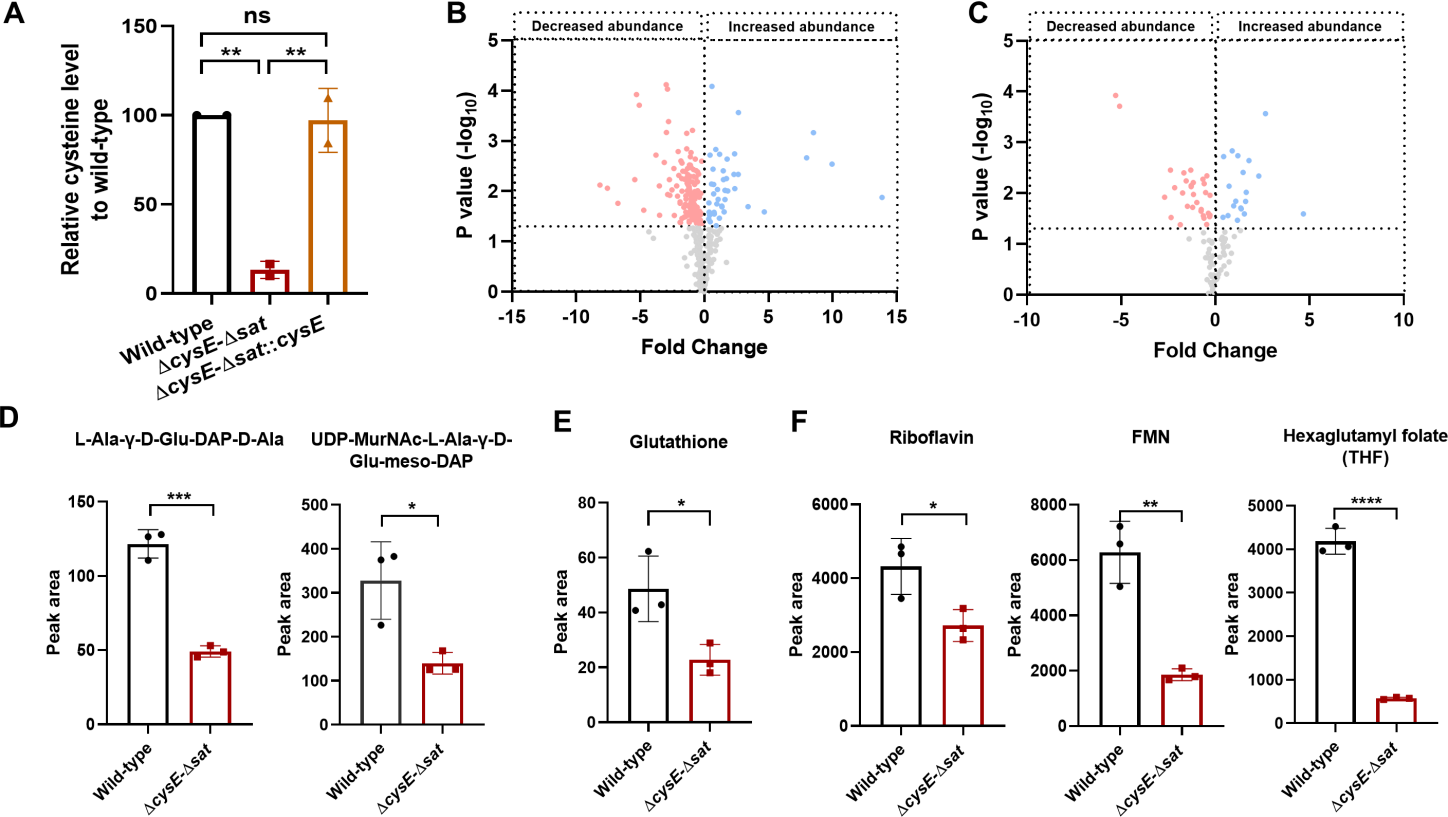
Disruption of cysteine biosynthesis leads to depletion of intracellular cysteine levels and metabolic dysregulation. (**A**) Relative intracellular cysteine level in the Δ*cysE*-Δ*sat* and Δ*cysE*-Δ*sat::cysE* compared to the wild type. Cells were grown till the mid-log phase, and cysteine concentration was assessed using the Sigma MAK255 kit according to the manufacturer’s protocol. The data represent values from two biological replicates. The values were normalized to CFU and then plotted as relative concentrations, with the wild type taken as the control. Statistical significance was measured using one-way ANOVA with multiple comparisons, where ** represents *P*-value < 0.01. Volcano plots depicting fold-change in the abundance of overall metabolites (**B**) and fatty acids and phospholipids (**C**), in the Δ*cysE-*Δ*sat* strain compared to the wild-type strain. Peak area of a few important metabolites belonging to the classes peptidoglycan precursors and recycling products (**D**), antioxidant (**E**), and vitamins and cofactors (**F**) in the wild-type and Δ*cysE-*Δ*sat* strains. The data represent the values from three biological replicates. Statistical significance was measured using a t-test, where **** represents *P*-value < 0.0001, *** represents *P*-value < 0.001, ** represents *P*-value < 0.01, and * represents *P*-value < 0.05.

Due to the disruption of cysteine biosynthesis, the Δ*cysE*-Δ*sat* strain relies on the uptake of cystine and cysteine from the medium. When grown in LB broth, the wild-type cells can both biosynthesize cysteine and uptake cystine from the medium, in contrast to the Δ*cysE-*Δ*sat* strain, which relies solely on the uptake system for growth. This difference also influences intracellular cysteine level, as the intracellular cysteine level in the Δ*cysE-*Δ*sat* strain was found to be significantly lower than that of the wild-type strain (Fig. 4A). Since cysteine is present in various proteins with diverse functions, including maintaining metabolic homeostasis, and the size of bacterial cells is significantly influenced by their metabolic state, we investigated the impact of disrupting cysteine biosynthesis on cellular metabolic homeostasis. We performed a metabolomic analysis comparing wild-type and Δ*cysE-*Δ*sat* strains grown in LB broth. Interestingly, our metabolomics analysis uncovered a link between cysteine biosynthesis and the intracellular levels of key metabolites, including peptidoglycan precursors, fatty acids, membrane phospholipids, and several essential vitamins and cofactors (Fig. 4B through F). Our analysis revealed reduced intracellular concentration of the peptidoglycan precursors UDP-N-Acetylmuramoyl-L-alanyl-D-gamma-glutamyl-meso-2,6-diamino-heptanedioate and peptidoglycan tetrapeptide (L-Ala-γ-D-Glu-DAP-D-Ala) in the Δ*cysE-*Δ*sat* strain. Notably, the intracellular level of the cofactors flavin mononucleotide (FMN), along with the precursor riboflavin (31), was also lower in the Δ*cysE-*Δ*sat* strain. FMN and flavin adenine dinucleotide (FAD), which is synthesized from FMN, are essential cofactors for multiple other enzymes involved in key metabolic pathways, including the tricarboxylic acid (TCA) cycle and the electron transport chain. Furthermore, we observed that the disruption of cysteine biosynthesis affects phospholipid homeostasis, as evidenced by altered intracellular levels of several phospholipids. The depletion of peptidoglycan precursors, essential for cell wall synthesis, along with disrupted phospholipid homeostasis, which may affect membrane biogenesis, and altered levels of riboflavin and FMN, crucial for metabolic processes, collectively influenced perturbations in cell size observed in cysteine biosynthesis mutants. Disruption of cysteine biosynthesis affected several other key metabolites, including glutathione, which is synthesized from cysteine and is crucial for mitigating oxidative stress. The intracellular levels of tetrahydrofolate (THF) and intermediates in vitamin B12 synthesis were also affected, indicating broader metabolic disruption. However, the virulence of the Δ*cysE-*Δ*sat* mutant remained unaffected, as observed in a murine pneumonia infection model, in which the lungs from mice infected with the wild type and the mutants showed comparable bacterial organ burdens (Fig. S6). This indicates that the uptake of cystine from the host sustained growth and fitness, further highlighting the metabolic resilience of *A. baumannii*, which retains the ability to establish infection despite metabolic disruption.

### Disruption of cysteine biosynthesis leads to a defect in cellular bioenergetics and oxidative stress management

**Fig 5:**
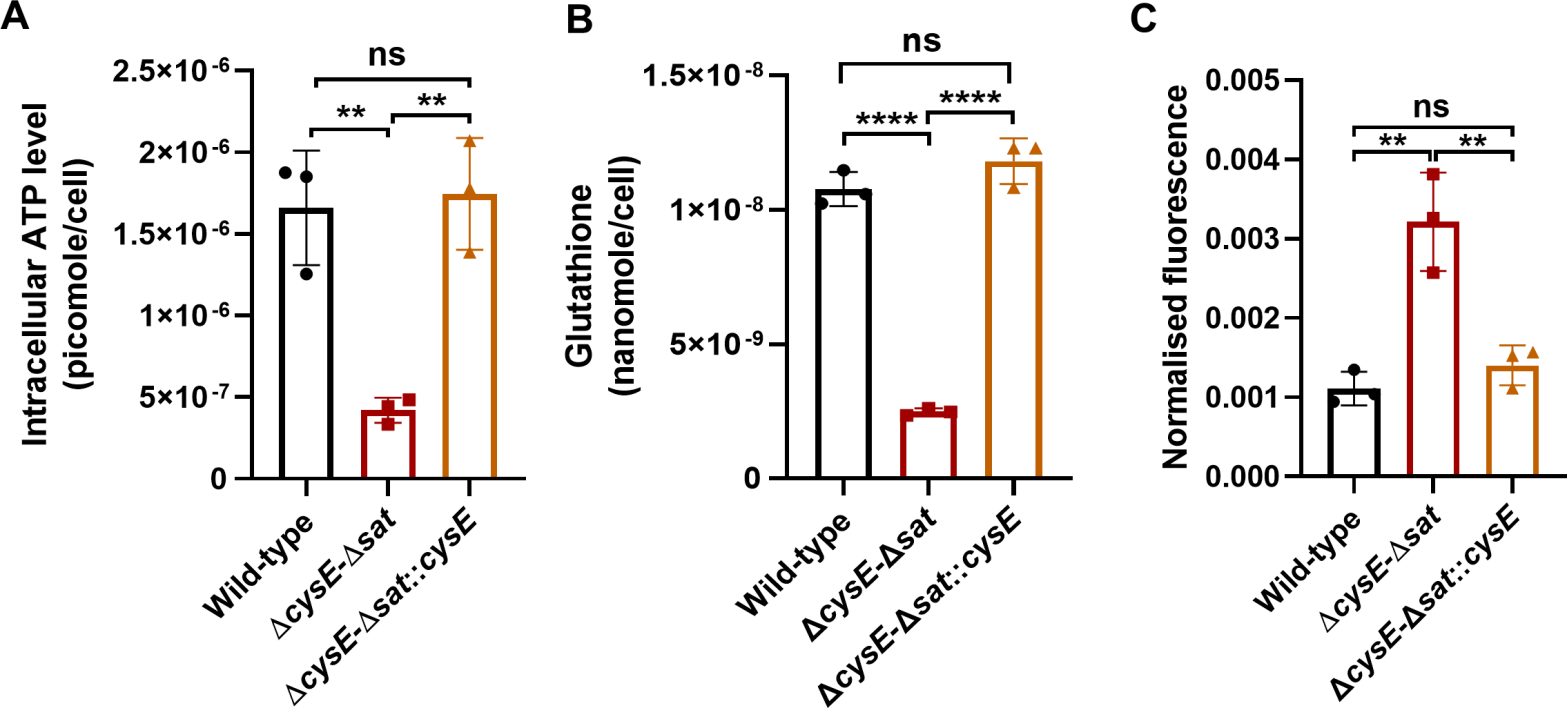
Disruption of cysteine biosynthesis leads to a defect in cellular bioenergetics and oxidative stress management. (**A**) Intracellular ATP content in wild-type, Δ*cysE-*Δ*sat,* and Δ*cysE-*Δ*sat::cysE* strains. The data represent values from three biological replicates. Statistical significance was measured using one-way ANOVA with multiple comparisons, where ** represents *P*-value < 0.01, and ns represents non-significant. (**B**) Intracellular glutathione content in wild-type, Δ*cysE-*Δ*sat,* and Δ*cysE-*Δ*sat::cysE* strains. The data represent values from three biological replicates. Statistical significance was measured using one-way ANOVA with multiple comparisons, where **** represents *P*-value < 0.0001, and ns represents non-significant. (**C**) Normalized fluorescence values representing intracellular ROS levels in wild-type, Δ*cysE-*Δ*sat,* and Δ*cysE-*Δ*sat::cysE* strains. The data represent values from three biological replicates. Statistical significance was measured using one-way ANOVA with multiple comparisons, where ** represents *P*-value < 0.01, and ns represents non-significant.

Our metabolomics data revealed depletion of vitamins and cofactors in the Δ*cysE-*Δ*sat* strain, along with depletion of other key metabolites. We therefore aimed to check whether this altered metabolome affects cellular bioenergetics by assessing intracellular ATP levels, a key indicator of cellular metabolic status. We found that intracellular ATP levels were significantly lower in the Δ*cysE-*Δ*sat* strain, indicating a defect in cellular bioenergetics (Fig. 5A). Our metabolomics analysis showed a depletion in intracellular glutathione levels in the Δ*cysE-*Δ*sat* strain, which we further confirmed using a secondary assay (Fig. 5B). Glutathione is the predominant low-molecular-weight (LMW) thiol in gram-negative bacteria and is a critical factor in bacterial resistance to oxidative stress (9) . Both cysteine and glutathione are involved in reducing nonphysiological disulfide bonds in cellular proteins formed during oxidative stress, thereby restoring their activity (9, 32). Furthermore, glutathione and cysteine are involved in S-glutathionylation and S-cysteinylation, respectively, reversible processes that protect cysteine residues in cellular proteins from oxidative stress-mediated irreversible damage (9, 33). Therefore, cysteine and glutathione are critical thiols in protecting cells from redox imbalance. Since both were depleted in the Δ*cysE-*Δ*sat* strain, we assessed intracellular reactive oxygen species (ROS) levels in cells grown in LB broth using a fluorescence-based assay. The Δ*cysE-*Δ*sat* strain exhibited a significant increase in intracellular ROS compared to the wild type, indicating a defect in oxidative stress mitigation (Fig. 5C).

### Disruption of cysteine biosynthesis promotes sensitivity to antibiotics *in vitro* and *in vivo*

**Fig 6:**
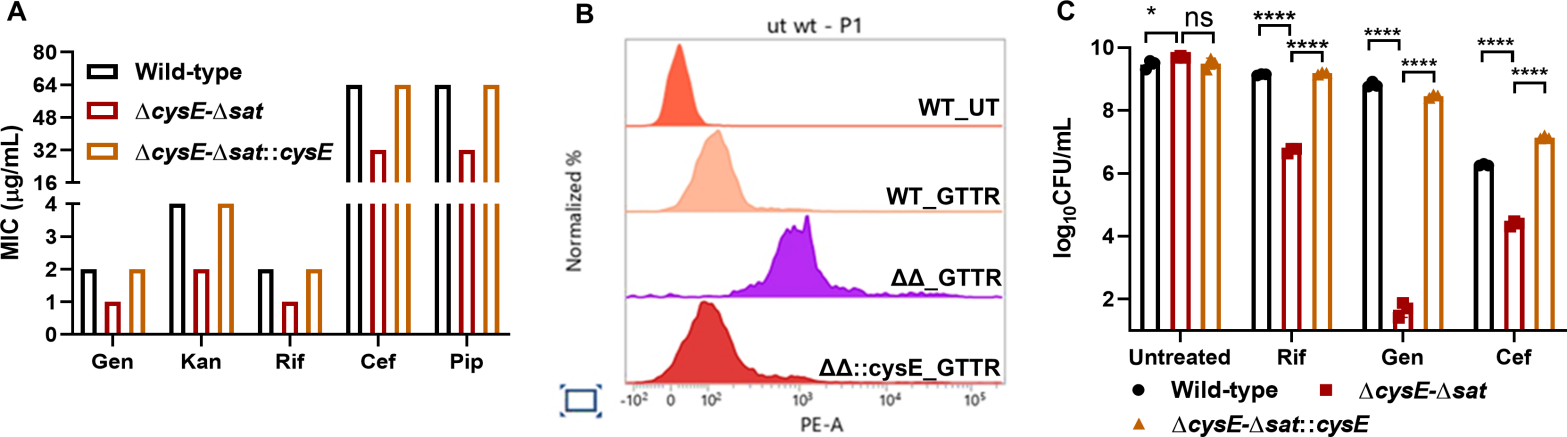
Disruption of cysteine biosynthesis leads to increased intracellular antibiotic accumulation and enhanced sensitivity. (**A**) Minimum inhibitory concentration (MIC) of indicated antibiotics for wild-type, Δ*cysE-*Δ*sat,* and Δ*cysE-*Δ*sat::cysE* strains determined by microbroth dilutions. Gen = Gentamicin, Kan = Kanamycin, Rif = Rifampicin, Cef = Cefotaxime, Pip = Piperacillin. (**B**) Gentamicin Texas Red (GTTR) accumulation assays representing the accumulation of gentamicin-Texas Red conjugate in wild-type, Δ*cysE-*Δ*sat,* and Δ*cysE-*Δ*sat::cysE* strains. WT_UT = wild-type untreated, WT_GTTR = wild-type treated with GTTR, ΔΔ_GTTR = Δ*cysE*-Δ*sat* treated with GTTR, ΔΔ::*cysE*_GTTR *=* Δ*cysE*-Δ*sat::cysE* treated with GTTR. (**C**) Endpoint CFU values representing survival of wild-type, Δ*cysE-*Δ*sat*, and Δ*cysE-*Δ*sat::cysE* strains after incubation with 1 µg/mL rifampicin, 1 µg/mL gentamicin, or 32 µg/mL cefotaxime under shaking conditions. The data represent values from three biological replicates. Statistical significance was measured using one-way ANOVA with multiple comparisons, where **** represents *P*-value < 0.0001, * represents *P*-value < 0.05, and ns represents non-significant.

**Fig 7:**
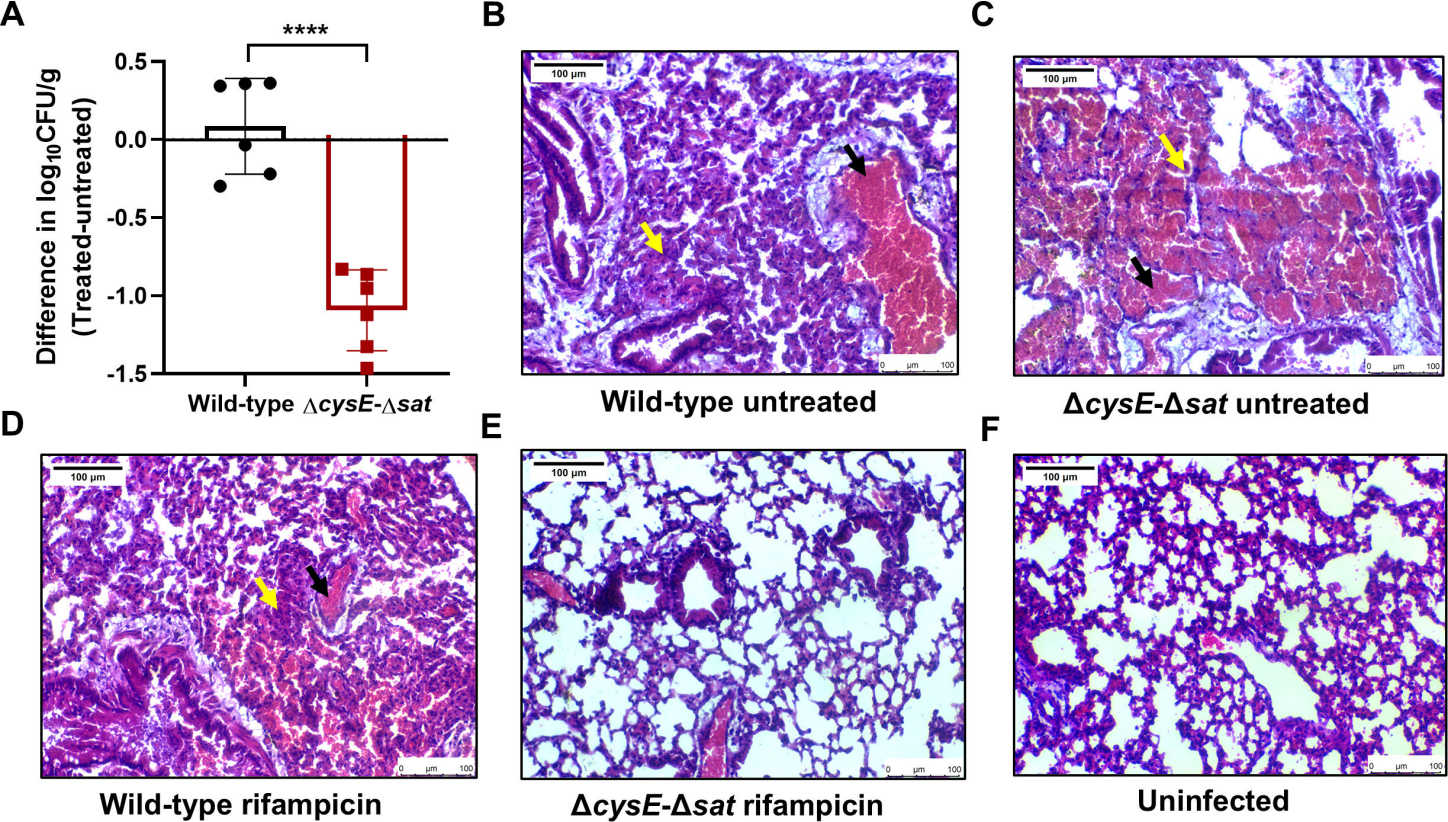
Disruption of cysteine biosynthesis leads to increased killing by rifampicin *in vivo.* (**A**) Differences in bacterial burden in the lungs of mice in the rifampicin-treated group compared to the untreated group. Following infection, the treatment group received rifampicin at a dose of 5 mg/kg body weight at 2, 14, and 26 hours. The organ burden was enumerated 36 hours post-infection, and the organ burden in the untreated group was subtracted from the treated group to indicate the difference. *n* = 6. Statistical significance was measured using a t-test, where **** represents *P*-value < 0.0001. (**B–F**) Representative histopathological images (hematoxylin and eosin staining; scale bar, 100 μm) of lung tissues. Lung tissues infected with wild type (**B**) and the Δ*cysE*-Δ*sat* (**C**) from untreated groups show significant loss of integrity of alveolar spaces, immune cell infiltration (yellow arrows), and hemorrhage (black arrows). Lung tissue from mice infected with wild type and treated with rifampicin (**D**) also shows similar damage. Lung tissue from mice infected with the Δ*cysE*-Δ*sat* and treated with rifampicin (**E**) shows markedly reduced damage with preserved alveolar structure. (F) Lung tissue from uninfected control with preserved alveolar architecture.

Disruption of cysteine biosynthesis led to depletion of peptidoglycan precursors and recycling products, as well as the intracellular pool of LMW thiols. The depletion of intracellular levels of peptidoglycan biosynthesis and recycling products indicates a negative impact on peptidoglycan biosynthesis and recycling. This likely arises as the key enzymes in these pathways, including MurA and LdtAB, require active-site cysteines. Defects in peptidoglycan recycling and folate metabolism have been shown to affect peptidoglycan precursor pools and to increase β-lactam sensitivity in *Caulobacter crescentus* and *Pseudomonas aeruginosa* (34, 35). On the other hand, cysteine is a precursor to LMW thiol glutathione, a critical component in cellular redox homeostasis. Antibiotics like aminoglycosides and rifampicin are known to induce ROS within cells as a secondary mechanism of killing, in addition to targeting their canonical targets (36, 37). Additionally, smaller cells, owing to their higher surface-to-volume ratio, tend to exhibit higher intracellular antibiotic concentrations, and dysregulation in cellular bioenergetics also affects the exclusion of antibiotics from cells by active efflux (38, 39). Therefore, we assessed whether the metabolic dysregulation observed in the Δ*cysE*–Δ*sat* strain also influences its sensitivity to those antibiotics. In line with these, we found the Δ*cysE*–Δ*sat* strain to exhibit enhanced sensitivity to antibiotics belonging to β-lactam, aminoglycosides, and rifampicin (Fig. 6A). In a gentamicin-texas red accumulation assay, the Δ*cysE*–Δ*sat* strain was also found to accumulate more gentamicin-texas red (GTTR) compared to the wild type (Fig. 6B). We further assessed cell survival in the presence of antibiotics under shaking conditions by incubating cells with the same initial inoculum at the indicated antibiotic concentrations for 20 hours, followed by CFU enumeration. The Δ*cysE*–Δ*sat* strain showed a significant reduction in cell viability in the presence of the antibiotics (Fig. 6C). To further assess whether Δ*cysE-*Δ*sat* also exhibits heightened sensitivity *in vivo* during an infection, we infected mice intranasally with the wild-type and Δ*cysE-*Δ*sat* strains. The animals were then divided into a rifampicin-treated group and an untreated group. The treatment group received rifampicin treatment at 2, 14, and 26 hours post-infection at a dose of 5 mg/kg body weight. At 36 hours post-infection, the animals were sacrificed, and the bacterial burden in the lungs was checked. The difference in bacterial burden following rifampicin treatment was calculated by subtracting the lung bacterial burden (Log10 CFU/g) in the untreated group from that of the rifampicin-treated group. Animals infected with the Δ*cysE-*Δ*sat* strain showed significantly higher reduction in bacterial burden upon rifampicin treatment compared to animals infected with the wild type (Fig. 7A). Furthermore, the lung tissues were stained with hematoxylin and eosin (H&E) to visualize the damage caused by infection. Lung tissues from mice infected with the wild-type strain, in both treated and untreated groups, as well as those from mice infected with the Δ*cysE*-Δ*sat* strain in the untreated group, exhibited significant damage to the alveolar architecture. This was characterized by immune cell infiltration (yellow arrows), hemorrhage (black arrows), and disruption of alveolar integrity (Fig. 7B through D). In contrast, lung tissues from mice infected with the Δ*cysE*-Δ*sat* strain in the treated group showed minimal inflammatory infiltration and largely preserved alveolar structure (Fig. 7E). This confirms that the Δ*cysE-*Δ*sat* strain also exhibits heightened sensitivity to rifampicin *in vivo*. Collectively, our findings indicate that the disruption of cysteine biosynthesis exerts a profound effect on cellular metabolism, leading to enhanced antibiotic susceptibility *in vitro* and *in vivo*.

### Disruption of cysteine biosynthesis leads to upregulation of genes involved in methionine biosynthesis, sulfate accumulation, and cystine transport

**Fig 8:**
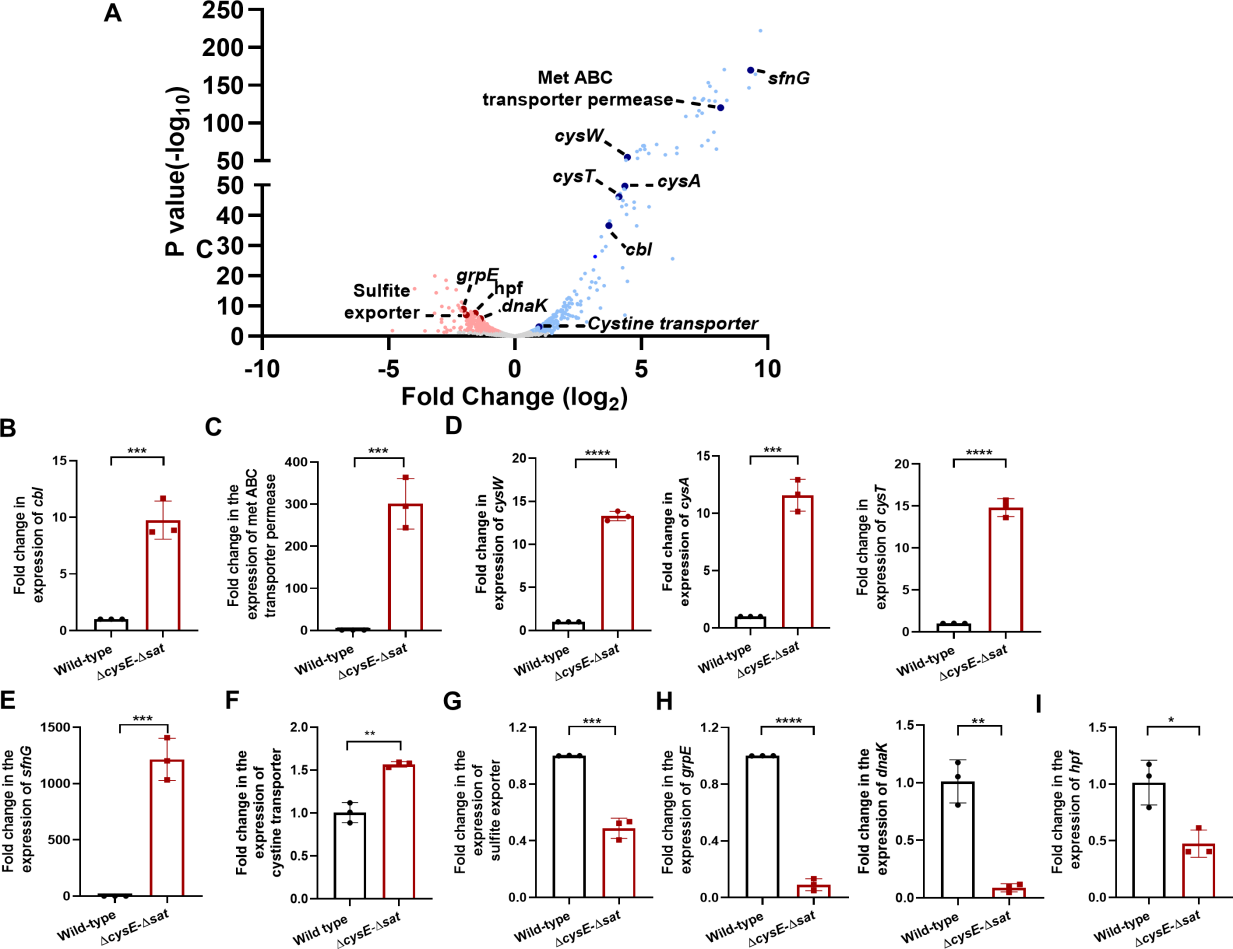
Disruption of cysteine biosynthesis leads to alterations in transcript abundance of several genes, including genes involved in methionine biosynthesis and uptake, sulfate accumulation, organosulfur metabolism, cystine transport, etc. (**A**) A volcano plot representing the differential expression of genes in the Δ*cysE-*Δ*sat* strain compared to the wild-type strain, grown in LB broth, with some of the genes involved in methionine uptake, organosulfur utilization, and sulfate accumulation highlighted. The data represent values from two biological replicates. Some of the genes involved in (**B**) transcriptional regulation of sulfate accumulation, (**C**) methionine transport, (**D**) sulfate accumulation, (**E**) organosulfur metabolism, (**F**) cystine transport, (**G**) sulfite export, (**H**) chaperone activity, and (**I**) ribosome sequestration were validated through RT-PCR. The data represent values from three biological replicates. Statistical significance was measured using a t-test, where **** represents *P*-value < 0.0001, *** represents *P*-value < 0.001, ** represents *P*-value < 0.01 and * represents *P*-value < 0.05.

When grown in LB broth, the cells rely on cystine uptake in the absence of the biosynthetic arm. To identify genes that support growth in the absence of cysteine biosynthesis, we performed RNA-seq analysis comparing gene expression profiles of the Δ*cysE-*Δ*sat* strain, which cannot synthesize cysteine, with those of the wild-type strain. The transcript abundance of several genes was altered in the Δ*cysE*-Δ*sat* strain compared to the wild type (Fig. 8A). These changes were further validated for a subset of genes by RT-PCR. The *cbl* regulator (Fig. 8B), along with genes involved in methionine transport (Fig. 8C), sulfate accumulation (Fig. 8D), metabolism of organosulfur compounds (Fig. 8E), and cystine transport (Fig. 8F), was found to be upregulated in the Δ*cysE-*Δ*sat* strain. Such changes in the cellular transcriptome are indicative of sulfur starvation. In addition, sulfite exporter, chaperone *dnaK*, nucleotide exchange factor *grpE*, and hibernation-promoting factors were found to be downregulated in the Δ*cysE-*Δ*sat* strain, among others (Fig. 8G through I). What was of particular interest was the cystine transporter (KZA74_05235), which was found to be upregulated in the Δ*cysE-*Δ*sat* strain, although the upregulation was modest (Fig. 8A and F). We hypothesize that this cystine transporter is involved in the uptake of cystine from the medium, thereby supporting growth in the absence of the biosynthesis arm. To investigate the importance of this transporter, we generated deletion mutants of the transporter in wild-type and Δ*cysE* backgrounds and annotated them Δ*05235* and Δ*cysE*-Δ*05235*, respectively. The mutants showed no growth or *in vivo* fitness defects compared to wild type, indicating that cysteine biosynthesis and uptake are functionally redundant for *A. baumannii* fitness (Fig. S7A through C).

### Deletion of the cystine transporter in the Δ*cysE-*Δ*sat* strain leads to synthetic lethality and *in vivo* fitness impairment

**Fig 9:**
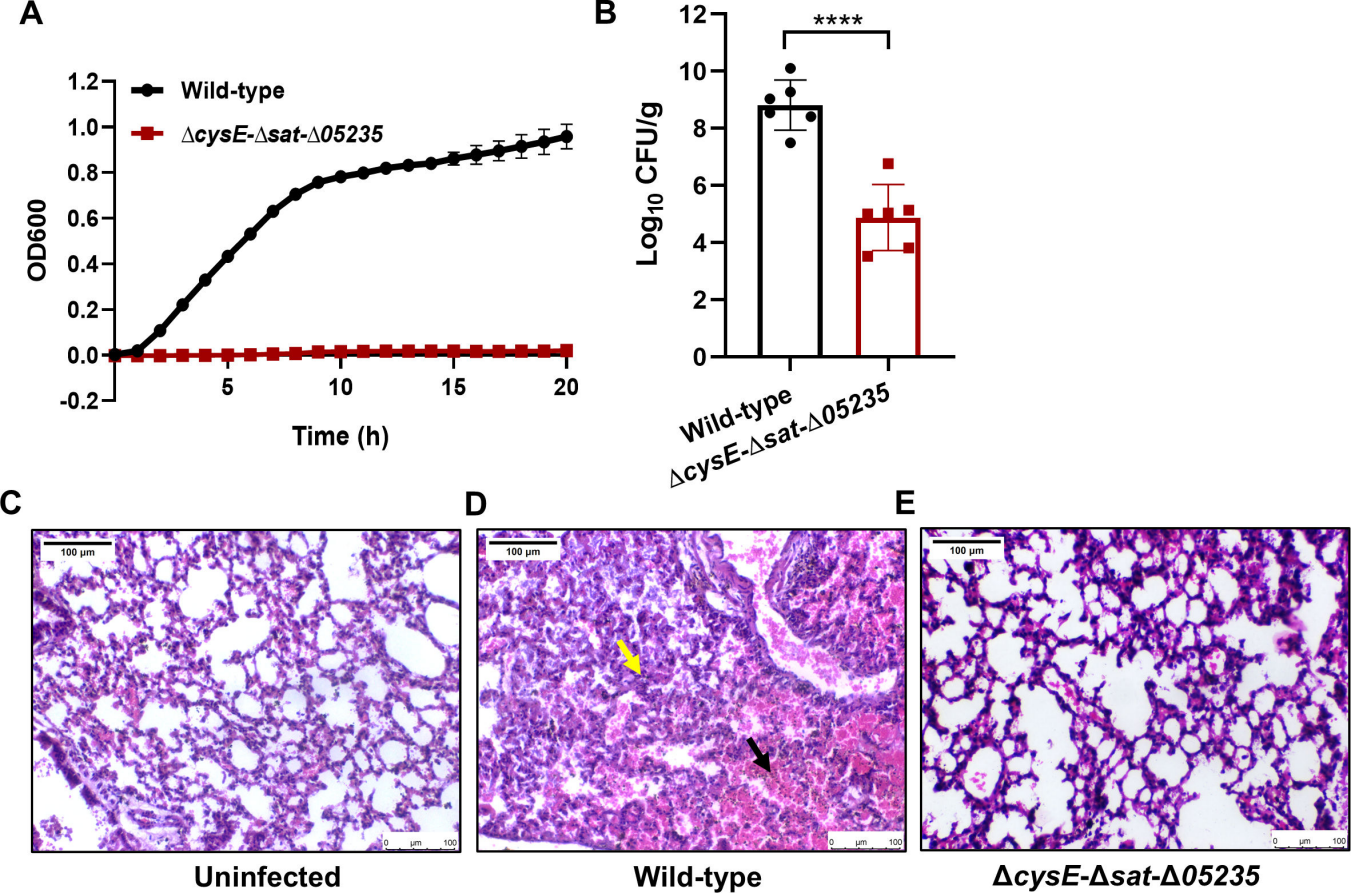
Disruption of cysteine biosynthesis and cystine transport leads to synthetic lethality and *in vivo* fitness impairment. (**A**) Growth profile analysis of wild-type and the triple deletion mutant of *cysE*, *sat*, and cystine transporter (Δ*cysE*-Δ*sat*-Δ*05235*) in LB broth. Each point represents the mean of three values with SD shown as error bars. (**B**) Enumeration of bacterial organ burden in mice lungs infected with the wild-type and Δ*cysE-*Δ*sat-*Δ*05235* strains at 36 hours post-infection. *n* = 6. Statistical significance was measured using a t-test. **** represents *P*-value < 0.0001. Representative tissue histopathology images (scale bar 100 μm) of mouse lungs (hematoxylin and eosin stained) of uninfected control (**C**) and mice infected with wild type (**D**) and Δ*cysE-*Δ*sat-*Δ*05235* (**E**). Lung from wild-type-infected mice shows significant loss of integrity of alveolar spaces, immune cell infiltration (yellow arrows), and hemorrhage (black arrows), while the lung from the Δ*cysE-*Δ*sat-*Δ*05235*-infected mice shows preserved alveolar structure and minimal damage.

Our initial attempts to generate a triple deletion mutant of *cysE*, *sat*, and cystine transporter were unsuccessful, leading us to hypothesize that this may be due to synthetic lethality, as the annotated cystine transporter is likely the predominant, if not the sole, cystine transporter, and its loss in cysteine auxotrophs would essentially deprive the cells of cysteine. However, we found that Δ*cysE-*Δ*05235* showed growth enhancement when supplemented exogenously with a high concentration of cysteine, compared to growth in M9-succinate alone, indicating that the mutant is able to uptake cysteine, possibly through low-affinity or non-specific transporters (Fig. S8A and B). Therefore, we retried making the triple deletion mutant and screened the recombinants on an LB agar plate containing the selective antibiotic and exogenously added cysteine. Following successful screening, we assessed the growth profile of the wild type and the triple deletion mutant, Δ*cysE-*Δ*sat-*Δ*05235*, in LB broth and LB broth supplemented with cysteine. The mutant failed to grow in LB broth, thus supporting our previous hypothesis of synthetic lethality (Fig. 9A and Fig. S9). We further assessed the fitness of the Δ*cysE-*Δ*sat-*Δ*05235* strain in a murine pneumonia infection model. Mice infected with the mutant exhibited significantly lower bacterial burden in lungs compared to those infected with the wild type, indicating significant attenuation in virulence (Fig. 9B). Furthermore, hematoxylin and eosin–stained lung sections from mice infected with the wild-type strain exhibited significant damage to lung tissue, characterized by immune cell infiltration (yellow arrows), hemorrhage (black arrows), and disruption of alveolar architecture (Fig. 9D). In contrast, lungs from mice infected with the Δ*cysE*-Δ*sat*-Δ*05235* strain showed markedly reduced damage, with minimal infiltration of immune cell, minimal hemorrhage and largely preserved alveolar structure (Fig. 9E).

## DISCUSSION

*A. baumannii* is a critical nosocomial pathogen and is a serious threat to healthcare settings. The ability to thrive on various carbon and nitrogen sources, supported by a complex network of metabolic pathways, enables *A. baumannii* to establish infections in diverse host niches (1). However, the metabolic circuits involving the biosynthesis, uptake, and interconversion of metabolites are inherently complex, and their implication in pathophysiological fitness remains largely unexplored. In light of the growing resistance to conventional antibiotics, there is increasing interest in identifying novel drug targets within central metabolism, particularly in pathways lacking homology to mammalian systems for therapeutic interventions. However, pathogens often exploit alternative pathways to ensure the supply of critical metabolites; thus, finding novel pathways for therapeutic interventions warrants a thorough systematic characterization.

Cysteine is a sulfur-containing amino acid that is present in the active sites of enzymes involved in diverse functions, including peptidoglycan biosynthesis and recycling, vitamin biosynthesis, and oxidative stress mitigation. However, a comprehensive understanding of the role of cysteine biosynthesis and uptake dynamics in maintaining the intracellular cysteine pool and its importance in overall cellular metabolism, antibiotic stress survival, and pathophysiological fitness in *A. baumannii* has been lacking. In *M. tuberculosis*, *cysE* is important for survival under oxidative stress, *in vivo* fitness, and survival under antibiotic stress (20). Notably, *M. tuberculosis* possesses alternate routes for the supply of required cysteine. This includes a reverse transsulfuration pathway, and cysteine uptake systems (40, 41). The defect caused by the deletion of *cysE* thus highlights the complexities of metabolic networks, including partial redundancy and the specific contributions of pathways toward maintaining the supply of critical metabolites. In *Listeria monocytogenes*, the cysteine biosynthesis pathway does not play a significant role in virulence, and the pathogen mostly relies on uptake to meet intracellular cysteine demand (42). These two features in *L. monocytogenes* and *M. tuberculosis* further highlight differences in metabolic pathways across bacterial species, underscoring the need for systematic, species-specific studies.

In *A. baumannii*, we found that cysteine biosynthesis is mediated by two serine acetyltransferases, CysE and SAT. To assess their individual contributions to cysteine biosynthesis and pathophysiological fitness, we constructed individual and double deletion mutants of *cysE* and *sat*. For *cysE*, the upstream RNA methyltransferase gene is transcribed from the same strand, and the last eight nucleotides of the RNA methyltransferase open reading frame (ORF) overlap with the *cysE* ORF. The downstream ABUW_2363 family tetratricopeptide repeat lipoprotein gene is also encoded on the same strand as *cysE*, with a 103-nucleotide gap between the two ORFs. Therefore, complete deletion of the cysE ORF could potentially affect the expression of the downstream gene and also lead to truncation of the upstream gene ORF. To avoid this, we generated a partial deletion mutant of *cysE* by removing 99 nucleotides spanning positions 58 to 156 relative to the AUG start codon. We also confirmed the absence of polar effect on the upstream and downstream genes by RT-PCR (data not shown). Similarly, for *sat*, we constructed a partial deletion mutant by removing 679 nucleotides spanning positions 4–682 relative to the AUG start codon to avoid any potential impact on the downstream gene expression.

Deletion of *cysE* resulted in a significant growth defect in a cysteine defecient medium (M9-succinate) , whereas deletion of *sat* did not. These findings indicate that CysE is the predominant serine acetyltransferase. Deletion of both genes rendered *A. baumannii* incapable of growing in M9-succinate, confirming that the CysE and SAT-mediated biosynthetic arm constitutes the sole cysteine biosynthesis pathway in *A. baumannii*. Exogenous supplementation with cysteine or cystine restored growth of the mutant, confirming that the observed growth attenuation was due to a defect in cysteine biosynthesis. For growth in a complex medium containing cystine, the cysteine auxotroph Δ*cysE*-Δ*sat* strain relies on a cystine transporter that shares homology with the cation-cystine symporter of *Pseudomonas aeruginosa*. This highlights the metabolic complexities of *A. baumannii*, in which the pathogen employs multiple partially redundant pathways to meet its intracellular cysteine requirement. However, the uptake system cannot fully compensate for the loss of the biosynthesis arm, and the Δ*cysE-*Δ*sat* strain exhibits a significant depletion of the intracellular cysteine pool. Our metabolomics analysis revealed alterations in key metabolite levels upon disruption of cysteine biosynthesis. Notably, peptidoglycan precursors, vitamins and cofactors, and antioxidant compounds were depleted. Moreover, the intracellular level of several fatty acids and phospholipids was altered, with both an increased and decreased abundance observed in the Δ*cysE-*Δ*sat* strain. The metabolic changes further led to dysregulation of cellular bioenergetics, elevation of intracellular ROS, and increased sensitivity to antibiotics (Fig. S10). Deletion of both serine acetyltransferases also altered the cellular transcriptome. Notably, genes involved in methionine biosynthesis and uptake, including methionine synthase and methionine ABC transporters, were upregulated in the Δ*cysE-*Δ*sat* strain. Sulfate transporter genes, *cysTWA*, which facilitate sulfate accumulation for the synthesis of H₂S, a key sulfur donor in cysteine biosynthesis, were also upregulated. Furthermore, genes involved in the assimilation of sulfur from organosulfur compounds, such as *sfnB* family sulfur acquisition oxidoreductases and dimethylsulfone monooxygenase *sfnG*, were upregulated in the Δ*cysE-*Δ*sat* strain. Such changes, induced by the deletion of the cysteine biosynthesis pathway, are consistent with a sulfur starvation response. Interestingly, the *cbl* regulator was also upregulated in the Δ*cysE-*Δ*sat* strain, while there was no significant change in expression of *gigC*, indicating the possible involvement of Cbl in regulating sulfate starvation response in this pathogen. Furthermore, transcripts of genes involved in sulfite export, chaperone activity, such as *dnaK* and nucleotide exchange factor *grpE*, were depleted in the Δ*cysE-*Δ*sat* strain. However, despite these defects in metabolic homeostasis and cellular bioenergetics, the Δ*cysE*-Δ*sat* strain did not exhibit a fitness defect in a murine pneumonia infection model. Earlier studies using a transposon mutant library suggested that cysteine biosynthesis may be important for *A. baumannii* AB5075 for establishing infection in *Galleria mellonella*. However, the study was conducted using a different strain of *A. baumannii* and employed an infection model in which host larvae were challenged with the entire mutant library. In such a competitive setting, individual mutants had to compete against thousands of others, effectively representing a competition-based assay rather than a direct assessment of each mutant’s contribution to virulence in isolation (43). This further highlights the importance of systematic characterization, including the construction of individual deletion mutants and *in vivo* fitness assays, to ascertain the role of a pathway in the pathophysiological fitness of a pathogen. Such functional redundancy between the biosynthesis and uptake mechanisms is also observed in *Salmonella Typhimurium* for methionine, another sulfur-containing amino acid (16). Further deletion of the cystine transporter in the Δ*cysE-*Δ*sat* strain led to synthetic lethality and fitness attenuation in a murine pneumonia infection model.

Our study demonstrates the metabolic plasticity of *A. baumannii*, suggesting that, in the absence of the cysteine biosynthetic arm, the bacterium may employ an unidentified regulatory network to sense intracellular cysteine levels and reallocate it to critical pathways to sustain growth and virulence. This aspect of bacterial metabolic regulatory networks remains largely uncharacterized and represents a promising area for further investigation, with the potential to uncover master regulators that may serve as effective targets for antimicrobial therapy. Our work also provides mechanistic insights into how the intracellular cysteine pool is maintained through the coordinated activities of the serine acetyltransferases and the cystine transporter, and how disruption of this pool perturbs metabolic homeostasis, thereby enhancing antibiotic susceptibility. Our work further underscores that disrupting metabolic homeostasis by inhibiting cysteine biosynthesis can be an effective strategy to curb *A. baumannii* infections, particularly when combined with conventional antibiotics. Considering the increasing emergence of resistance to conventional antibiotics, combination therapies present a promising strategy. In particular, coupling conventional drugs with inhibitors targeting cysteine biosynthesis could effectively curb infections caused by *A. baumannii*. Such an approach will essentially enhance the efficacy of existing antibiotics by compromising the bacterium’s metabolic homeostasis and stress response pathways, thereby sensitizing it to antibiotic treatment. Furthermore, our work shows that inhibiting cysteine biosynthesis and cystine transport results in synthetic lethality, attenuating growth of the pathogen and effectively compromising its fitness within the host. This finding suggests that a combinatorial approach employing inhibitors targeting both cysteine biosynthesis and cystine uptake could serve as an effective strategy to mitigate *A. baumannii* infections.

## MATERIALS AND METHODS

### Bacterial strains and culture conditions

All the bacterial strains and plasmids used in this work are listed in Tables S1 and S2, respectively. Details on culture conditions are provided in the supplemental material.

### Cloning, expression and purification of recombinant enzymes

The genes were cloned into the pET28c vector, and the *E. coli* BL21DE3 strain was used for protein expression. The recombinant proteins were purified using Ni-NTA affinity chromatography as described previously (44) with some modifications. The detailed methodology is described in the supplemental material.

### Assessment of the enzymatic activity of purified proteins

The activity of the recombinant proteins was assessed following the procedure as described previously (26) with some modifications. The detailed methodology is described in the supplemental material.

### RNA-Seq analysis

Overnight-grown cells were inoculated into fresh LB broth with a 0.1% inoculum and grown to mid-log phase, and harvested at a time when they had comparable CFU/mL. The detailed methodology is described in the supplemental material.

### Construction of deletion mutants

The deletion mutants were constructed in *A. baumannii* ATCC17978 UN strain (NCBI reference seq. CP079931.1) using a one-step chromosomal recombineering approach described previously (27, 28). The detailed methodology is described in the supplemental material.

### Scanning electron microscopy

Overnight-grown cells were inoculated into fresh LB broth with a 0.1% inoculum and grown to mid-log phase for SEM imaging. The detailed methodology is described in the supplemental material.

### Assessment of intracellular cysteine level

Overnight-grown cells were inoculated into fresh LB broth with a 0.1% inoculum, grown to mid-log phase, and harvested for cysteine quantification. The assay was performed according to the manufacturer’s protocol (MAK255, Sigma), and the detailed methodology is described in the supplemental material.

### Metabolomic analysis of wild-type and Δ*cysE-*Δ*sat* strains

Overnight-grown cells were inoculated into fresh LB broth with a 0.1% inoculum, grown to mid-log phase, and harvested for metabolomics analysis. The detailed methodology is described in the supplemental material.

### Assessment of intracellular ATP levels

Overnight-grown cells were inoculated into fresh LB broth with a 0.1% inoculum and grown to mid-log phase. Intracellular ATP levels were measured using BacTiter-Glo. The detailed methodology is described in the supplemental material.

### Assessment of intracellular glutathione levels

Overnight-grown cells were inoculated into fresh LB broth with a 0.1% inoculum, grown to mid-log phase, and harvested for glutathione quantification. The assay was performed according to the manufacturer’s protocol (CS0260, Sigma), and the detailed methodology is described in the supplemental material.

### Assessment of ROS levels

Intracellular ROS was measured using the ROS-responsive dye 2′−7′-dichlorodihydrofluorescein diacetate (DCFH-DA). The detailed methodology is described in the supplemental material.

### Determination of MIC

MICs of the indicated antibiotics were determined by broth microdilution in Mueller-Hinton (MH) broth. Overnight-grown cells were subcultured into fresh MH broth and incubated until OD_600_ reached 0.3. The cultures were diluted to ~10^5^ CFU/mL and added to plates containing antibiotic dilutions. The plates were incubated for 18 hours at 37°C under static conditions, and the OD_600_ was measured using a plate reader (Agilent).

### Gentamicin Texas red accumulation assay

The assay was performed as previously described (45) with some modifications. The detailed methodology is described in the supplemental material.

### Growth assessment in the presence of antibiotics

Cells were grown overnight in MH broth, then subcultured into fresh MH broth and incubated until OD_600_ reached 0.3. The cultures were diluted to ~10^5^ CFU/mL and incubated for 20 hours at 37°C and 180 rpm shaking with or without the indicated antibiotics. The CFU at the endpoint was enumerated by serial dilution and spreading on LB agar plates.

### Mice pneumonia infection model

All animal experiments under protocol BT/IAEC/2017/05 and IAEC/2025/01 were reviewed and approved by the Institute Animal Ethics Committee (IAEC) of the Indian Institute of Technology, Roorkee. The detailed methodology is described in the Supplemental material.

## Data Availability

The raw data for the RNA-seq performed with the wild-type and ΔcysE-Δsat have been submitted to NCBI SRA with an ID.PRJNA1335821 and BioSample accessions: SAMN52024188, SAMN52024189, SAMN52024190, and SAMN52024191. Metabolomics raw data have been submitted to the Metabolomics Workbench with DOI https://dx.doi.org/10.21228/M85G1N . The reviewers' link to the datasetdata set is https://dev.metabolomicsworkbench.org:22222/data/DRCCMetadata.php?Mode=Study&StudyID=ST004026&Access=JfcZ1024 This article contains supporting information (Sett et al. 2024, Verma et al. 2020, Mino et al. 1999, Andrews et al. 2010, Chen et al. 2018, Liao et al. 2014, Love et al. 2014, Tucker et al. 2014, Bhowmik et al. 2025, Coppens et al. 2020).

## SUPPLEMENTAL MATERIAL

Supplemental material mbio.00842-26-s0001.pdf Supporting experimental procedures (S1-S14), Tables S1 to S4, and Figures S1-S10.

ASM does not own the copyrights to Supplemental Material that may be linked to, or accessed through, an article. The authors have granted ASM a non-exclusive, world-wide license to publish the Supplemental Material files. Please contact the corresponding author directly for reuse.
