# Supplementary material for: Disruption of cysteine metabolism leads to synthetic lethality and in vivo fitness impairment in Acinetobacter baumannii
Source: mBio. 2026 Jun 15;17(7):e00842-26. doi: 10.1128/mbio.00842-26 (PMC13344012; doi:10.1128/mbio.00842-26)
Supplement: Supplemental material — Supporting experimental procedures (S1-S14), Tables S1 to S4, and Figures S1-S10. [file mbio.00842-26-s0001.pdf]

Disruption of cysteine metabolism leads to synthetic lethality and *in vivo* fitness impairment in *Acinetobacter baumannii*

Avik Pathak[1], Snehlata Saini[1], Ranjana Pathania[1,2#]

[1]Department of Biosciences and Bioengineering, Indian Institute of Technology Roorkee, Roorkee, Uttarakhand, India

[2]Centre of Excellence in Disaster Mitigation and Management, Indian Institute of Technology Roorkee, Roorkee, Uttarakhand, India

[#]Address correspondence to Ranjana Pathania, ranjana.pathania@bt.iitr.ac.in

List of materials included:

Supporting experimental procedures

S1: Bacterial growth and culture conditions……………………………………………………..S2

S2. Cloning, expression, and purification of recombinant enzymes………………………..S2

S3: Assessment of the enzymatic activity of annotated serine acetyltransferases……….S3

S4: Assessment of the enzymatic activity of cysteine synthases……………….................S3

S5: Construction of deletion mutants and complemented strain……………………...…S4

S6: RNA-seq experiment……………………………………………………………………..S4

S7: Scanning electron microscopy…………………………………………………………..S5

S8: Assessment of intracellular cysteine level………………………………………………..S5

S9: Metabolomics analysis………………………………………………………………..…S5

S10: Assessment of intracellular ATP levels…………………………………………………..S5

S11: Assessment of intracellular glutathione levels…………………………………………S6

S12: Assessment of ROS level……………………………………………………….………..S6

S13: Gentamicin Texas red accumulation assay……………………………………………..S6

S14: Murine pneumonia infection model………………………………………………..…S6

Supporting Tables

Table S1: Strains used in this study………………………………………………………......S7

Table S2: Plasmids used in this study……………………………………………………......S8

Table S3: List of primers used in this study…………………………………………….…S8

Table S4: List of genes showing differential abundance in Δ*cysE*-Δ*sat* ………………. S12

Supporting Figures

Figure S1……………………………………………………………………..………………S17

Figure S2……………………………………………………………………………S18

Figure S3……………………………………………………………………………S18

Figure S4……………………………………………………………………………S19

Figure S5……………………………………………………………………………S20

Figure S6……………………………………………………………………………S21

Figure S7……………………………………………………………………………S21

Figure S8……………………………………………………………………………S22

Figure S9……………………………………………………………………………S22

Figure S10……………………………………………………………………………S23

Supporting references…………………………………………………………S23

Supporting experimental procedures:

S1: Bacterial growth and culture conditions

For growth profile analysis in cysteine-deficient defined medium, the *Acinetobacter baumannii* ATCC 17978 UN wild-type and mutant strains were grown in M9 minimal salts supplemented with succinate (20 mM). For growth profile analysis in the presence of exogenous cysteine and cystine, M9-succinate medium was supplemented with 200 µM of each. To prepare cells for SEM imaging and metabolomics analysis, cells were grown in LB broth. For the construction of a triple deletion mutant of *cysE*, *sat,* and the cystine transporter (Δ*cysE*-Δ*sat*-Δ*05235*), the recombinants were selected on LB agar containing apramycin as a selective agent and 200 µM cysteine. The growth profile analysis of the wild-type and the Δ*cysE*-Δ*sat*-Δ*05235* strains was performed in LB broth and LB broth supplemented with 200 µM cysteine. In all cases, the cells were grown at 37°C, except for curing plasmids pAT02 and pAT03, where they were grown at 42°C to facilitate plasmid curing. For broth cultures, cells were always incubated with shaking at 180 rpm.

S2: Cloning, expression, and purification of recombinant enzymes:

The protein-coding genes from *A. baumannii* ATCC17978 were amplified and cloned into the pET28c vector between NdeI and XhoI sites, and the recombinant plasmids were then transformed into *E. coli* BL21DE3 strains for protein expression. The proteins were purified following the procedures described previously (1, 2) with modifications. Overnight grown *E. coli* BL21DE3 cells harbouring the recombinant pET28c (pET28c-*sat*, pET28c-*cysE*, pET28c-*cysM*, pET28c-cysK) plasmid were subcultured in fresh LB broth containing kanamycin (50µg/mL) and grown till the OD600 reached 0.6. The cells were then induced with 1 mM IPTG and incubated for 4 hours. The cells were then harvested and washed with buffer containing 50 mM Tris, 500 mM NaCl, 1 mM β-mercaptoethanol and 10% glycerol, pH 7.3.

For the purification of SAT, CysK and CysM, bacterial cells were resuspended in binding buffer (50 mM Tris, 500 mM NaCl, 1 mM β-mercaptoethanol, and 10% glycerol, pH 7.3). Cell lysis was performed using a French press at 15 kpsi for three cycles. The lysate was then subjected to centrifugation at 10,000 rpm for 1 hour, and the supernatant was filtered through a 0.4 µm filter. A Ni-NTA affinity column (His Trap FF column, 5 ml, Cytiva) was recharged with 100 mM $NiSO_4$ solution and then equilibrated with the binding buffer. The filtrate containing the protein was then loaded onto the column fitted in an AKTA pure system and

allowed to bind to the column at a flow rate of 1 mL/ min. Following binding, the column was washed with buffer containing 50mM imidazole (50 mM Tris, 500 mM NaCl, 1 mM β-mercaptoethanol, 10% glycerol, 50 mM imidazole, pH 7.3) and then the proteins were eluted with a gradient of elution buffer (50 mM Tris, 500 mM NaCl, 1 mM β-mercaptoethanol, 10% glycerol, 500 mM imidazole, pH 7.3). Eluted fractions were analysed by SDS-PAGE to identify those with the highest protein content. The selected fractions with the highest quantity of proteins were then concentrated using a 10 kDa molecular weight cut-off filter and then dialysed against a buffer containing 20 mM Tris, 200 mM NaCl, 10% glycerol, pH 7.3 to remove imidazole. The protein was stored at -80°C in the same buffer till the assays were performed.

For purification of CysE, the harvested cells were resuspended in buffer A (50 mM Tris, 500 mM NaCl, 1 mM β-mercaptoethanol, and 10% glycerol, pH 7.3). Cell lysis was performed using a French press at 15 kpsi for three cycles. The lysate was then subjected to centrifugation at 11,000 rpm for 30 minutes. The supernatant was discarded, and the pellet was resuspended with buffer B (50 mM Tris, 500 mM NaCl, 1 mM β-mercaptoethanol, 8 M Urea and 10% glycerol, pH 7.3). The resuspended solution was then centrifuged at 11000 rpm for 1 hour. The supernatant was filtered through a 0.4 μm filter. A Ni-NTA affinity column (His Trap FF column, 5 ml, Cytiva) was recharged with 100 mM $NiSO_4$ solution and then equilibrated with the binding buffer. The filtrate containing the protein was then loaded onto the column fitted in an AKTA pure system and allowed to bind to the column at a flow rate of 1 mL/ min. Following binding, the bound proteins were allowed to renature on the column. This was done by running a gradient of buffer A to gradually remove urea from the system. Following this, the column was washed with buffer containing 50mM imidazole (50 mM Tris, 500 mM NaCl, 1 mM β-mercaptoethanol, 10% glycerol, 50 mM imidazole, pH 7.3) and then the proteins were eluted with a gradient of elution buffer (50 mM Tris, 500 mM NaCl, 1 mM β-mercaptoethanol, 10% glycerol, 500 mM imidazole, pH 7.3). Eluted fractions were analysed by SDS-PAGE to identify those with the highest protein content. The selected fractions with the highest quantity of proteins were then concentrated using a 10 kDa molecular weight cut-off filter and then dialysed against a buffer containing 20 mM Tris, 200 mM NaCl, 10% glycerol, pH 7.3 to remove imidazole. The protein was stored at -80°C in the same buffer till the assays were performed.

S3: Assessment of the enzymatic activity of annotated serine acetyltransferases

The activity of the recombinant proteins was assessed following the procedure described by (3) with some modifications**.** The assay leveraged the absorbance properties of the thioester bond in acetyl-CoA, which exhibits absorption at 232 nm. The cleavage of this bond by serine acetyltransferase during the synthesis of O-acetylserine results in a decrease in absorbance. Purified enzymes at varying concentrations were incubated with 5 mM L-serine and 200 μM acetyl-CoA in reaction buffer (20 mM Tris, 200 mM NaCl, pH 7.3) at 30°C. The final reaction volume was 200 μL. Absorbance at 232 nm was recorded every 10 minutes for 2 hours. The absorbance values were normalised to those of the purified proteins, and a time vs. change in absorbance (ΔAbs_{232}) curve was plotted using GraphPad Prism 8. The slope (ΔAbs_{232}/min) was calculated in Prism 8 and plotted against enzyme concentration. The negative value of the slope represents decay in substrate quantity over time.

S4: Assessment of the enzymatic activity of cysteine synthases

The activity of the recombinant proteins was assessed following the procedure described by (3) with some modifications**.** Varying concentrations of purified proteins were incubated with

7.5 mM O-acetyl serine and 750 µM Na_2S in reaction buffer (20 mM Tris, 200 mM NaCl, pH 7.3) for 5 minutes at 25°C. The final reaction volume was 150 µL. Following incubation, the reaction was stopped using 75 µL 20% (w/v) Trichloroacetic acid (TCA) and 150 µL glacial acetic acid. For colour development, 300 µL of 25mg/mL ninhydrin prepared in an HCl: Acetic acid (60:40) solution was added. The solution was then heated at 100°C for 10 minutes to develop colour. Absorbance was measured at 560 nm, and an enzyme concentration (log_{10}) vs absorbance curve was plotted for individual enzymes.

S5: Construction of deletion mutants and complemented strain

The deletion mutants were constructed following the procedure described by (4, 5). The upstream and downstream regions of the target sequence to be deleted were cloned into the pUC18 vector, flanking an apramycin resistance marker. This marker, amplified from plasmid pMDIAI, contains FRT sites to facilitate its subsequent removal. For *cysE*, *sat*, and the cystine transporter (KZA74_05235), partial deletion mutants were constructed to avoid disruption of potential promoters of upstream or downstream genes. A 99 bp region was deleted from *cysE*, 679 bp from *sat* and 1238 bp from the cystine transporter. Potential promoters were predicted using the SAPPHIRE promoter prediction tool (6).

For partial deletion mutants, stop codons were also introduced in-frame with the start codon (AUG) to prevent translation of the remaining ORF. A 3X FLAG tag was introduced in frame after the stop codons to facilitate confirmation using anti-FLAG antibodies, if needed. The recombinant construct was then amplified using primers complementary to the upstream and downstream regions. A total of 5 µg of the gel-purified knock-out amplicon was transformed into *A. baumannii* electrocompetent cells carrying plasmid pAT02 via electroporation. To promote homologous recombination, the Rec_{AB} recombinase from pAT02 was induced with 5 mM IPTG, and transformants were selected on LB agar plates containing apramycin (30 µg/mL). Transformants were screened via PCR using primers that bind further upstream and downstream of the region to which the primers used to amplify the knockout amplicon bind.

Following confirmation, pAT02 was cured, and cells were transformed with pAT03, which expresses FLP recombinase and contains an ampicillin resistance marker. The recombinase was induced using 5 mM IPTG, and the transformants were selected on ampicillin-containing plates (200 µg/mL). To confirm the removal of the apramycin resistance cassette, colonies were streaked in parallel on ampicillin and apramycin plates. Colonies that failed to grow on apramycin plates were further verified by PCR using upstream and downstream primers, confirming the loss of the apramycin cassette. The plasmid pAT03 was further cured from the mutant strain. For genetic complementation of Δ*cysE*-Δ*sat*, a similar strategy was used. The truncated copy of *cysE* in the Δ*cysE*-Δ*sat* strain was replaced with a functional copy of *cysE* with an apramycin cassette attached to it for selection. The cassette was later removed using the method mentioned before.

S6: RNA-seq experiment

To assess the transcriptome of wild type and Δ*cysE*-Δ*sat* in a complex medium, the cells were grown overnight in LB broth. The overnight-grown cells were then subcultured in fresh LB broth and grown to mid-log phase, and harvested at a time when they had comparable CFU/mL. Total RNA was extracted and sent for RNA-seq analysis following DNase I treatment. The RNA-seq analyses were performed by miBiome Therapeutics. QC-passed samples were processed for library preparation. 1 µg of bead-purified RNA was depleted of bacterial rRNA using the NEBNext® rRNA Depletion Kit (Bacteria, E7850), following the manufacturer's protocol. The rRNA-depleted RNA was purified and used for cDNA library

construction with the NEBNext® Ultra II Directional RNA Library Prep Kit for Illumina (E7760L). Libraries were quantified using a Qubit fluorometer, and fragment size distribution was assessed with a High Sensitivity D1000 ScreenTape. The quality of the reads was assessed using FastQC v 0.11.3 (7) before proceeding with the downstream analysis. The reads were trimmed using Fastp v 0.20.1 (8), and the quality passed reads were mapped onto the reference transcriptome using bowtie2 and were quantified with *featureCounts* v2.0.1 (9) using the gff3 file of the reference genome. The DESeq2 (10) was used for differential expression analysis. Genes were filtered based on false discovery rate cut-off (FDR) < 0.05.

S7: Scanning electron microscopy

Overnight grown cells were inoculated in fresh LB broth with 0.1% inoculum and grown till the mid-log phase. The cells were harvested and washed twice with PBS and fixed overnight with formaldehyde (2%). The fixed cells were then washed again with PBS, spotted on small glass slides and then dehydrated through an ethanol gradient. The cells were then coated with gold particles and visualised using a ZEISS Gemini 560 FE-SEM. The morphometric analysis was performed using ImageJ software and plotted using GraphPad Prism 8.

S8: Assessment of intracellular cysteine level

Cells were grown overnight in LB. The cells were then inoculated into fresh LB broth with a 0.1% inoculum and grown to mid-log phase. Cells were harvested and washed twice with 1X PBS. Cells were then resuspended in 3 mL PBS and lysed using a French press at 20kPsi for 3 cycles. Following lysis, the lysates were centrifuged at 10000 rpm for 50 minutes at 4°C. The supernatants were collected and used for cysteine quantification with the Cysteine Assay Kit (MAK255) from Sigma. The blank-adjusted fluorescence values for the samples were normalised with CFU. The values were then represented relative to the wild type.

S9: Metabolomics analysis

Overnight-grown wild-type and Δ*cysE*-Δ*sat* cells were freshly inoculated in 1 L LB broth with 0.1% inoculum. The cells were allowed to grow till the mid-log phase, and cells were harvested at a time point where both the strains show comparable CFU/mL. Harvested cells were then washed with 1X PBS two times. Samples were prepared independently on three different days and were sent for metabolomic analysis. The metabolomic analysis was performed by Fountomics Life Sciences.

For sample preparation, 250 mg of the cell pellets were taken, resuspended with lysis buffer, and sonicated for an hour. After sonication, the samples were incubated at 4°C for 2 hours and then centrifuged at 8000 rpm for 20 minutes. The supernatant was collected, and 3 volumes of methanol: chloroform: water (10:35:5) were added to that and incubated at -20°C for 2-3 hours. Following incubation, the samples were centrifuged at 8000 rpm for 20 minutes, and the supernatant was dried under a SpeedVac. The dried samples were redissolved with 100 µL of 90% methanol-water solution and injected into the UPLC-QTOF (XEVO-G2XSQTOF) system. Detection of metabolites was performed under positive ionisation mode with a scan range of 50-3000 m/z.

S10: Assessment of intracellular ATP levels

To measure intracellular ATP levels, a luciferin/luciferase assay was performed. Overnight-grown cells were inoculated into fresh LB broth with a 0.1% inoculum and grown to mid-log phase. The cells were harvested and washed twice with PBS. The cells were incubated with

BacTiter-Glo™ as per the manufacturer's protocol in a 96-well round-bottom white plate (Corning), and the luminescence was recorded using a BioTek Synergy H1 (Agilent) plate reader. The luminescence values were normalised with CFU. A standard curve was prepared with known ATP concentration in the same assay to calculate ATP content per cell.

S11: Assessment of intracellular glutathione levels

Cells were grown overnight in LB. The cells were then inoculated into fresh LB broth with a 0.1% inoculum and grown to mid-log phase. Cells were harvested and washed twice with 1X PBS. Cells were then resuspended in 3 mL PBS and lysed using a French press at 20kPsi for 3 cycles. Following lysis, the lysates were centrifuged at 10000 rpm for 50 minutes at 4°C. The supernatants were collected, and glutathione content was assessed according to the manufacturer’s protocol (CS0260, Sigma). The values were normalised with CFU and compared with the standard curve prepared in the same assay to quantify glutathione content per cell.

S12: Assessment of ROS level

Intracellular ROS was measured using the ROS-responsive dye 2′− 7′-dichlorodihydrofluorescein diacetate (DCFH-DA). Briefly, overnight-grown cultures were inoculated into fresh LB broth with a 0.1% inoculum and grown until the mid-log phase. The cells were then harvested, washed twice with PBS, and resuspended in 1 mL of 1X PBS. The dye was added to a final concentration of 10 µM and incubated for 30 minutes at 37 °C. The cells were then washed twice, and fluorescence was measured using a BioTek Synergy H1 (Agilent) plate reader with 485 nm excitation and 530 nm emission, at 1-minute intervals. The fluorescence values were normalised with CFU and plotted using GraphPad Prism 8 software

S13: Gentamicin Texas red accumulation assay

Overnight-grown cultures were inoculated into fresh LB broth at 0.1% inoculum and grown to mid-log phase. The cells were then harvested by centrifugation and resuspended in fresh LB broth. The cells were divided into two groups: untreated and treated. Gentamicin-texas red conjugate was added to the treatment group at a final concentration of 0.5 µg/mL and incubated for 45 minutes at 37 °C under shaking conditions. The cells were then harvested, washed twice with 1X PBS, and resuspended in 200 µL 2% formaldehyde. The resuspended cells were then diluted by adding 800 µL of 1X PBS and analysed using a BD flow cytometer (BD FACSLyric) with an excitation laser at 496nm and a bandpass filter of 586/42 nm. 10000 events were captured.

S14: Murine pneumonia infection model

All animal experiments under protocol BT/IAEC/2017/05 and IAEC/2025/01 were reviewed and approved by the Institute Animal Ethics Committee (IAEC) of the Indian Institute of Technology, Roorkee. Mice aged 6–8 weeks were used for the study. Neutropenia was induced via intraperitoneal injection of cyclophosphamide (150 mg/kg body weight) prepared in sterile normal saline, administered on days 4 and 1 prior to infection. Anaesthesia was induced through intraperitoneal injection of 100 µL of a solution containing Ketamine (80 mg/kg body weight) and Xylazine (20 mg/kg body weight) prepared in sterile normal saline.

To prepare the inoculum, overnight-grown cells were freshly inoculated in LB broth with 0.1% inoculum and incubated till the mid-log phase. Cells were harvested and washed twice with normal saline and resuspended in normal saline to achieve a final concentration of 5×10^9 CFU/mL. Each mouse was infected intranasally with 20 µL of this suspension, delivering

$1×10^8$ CFU per animal. 36 hours post-infection, the mice were sacrificed, and the lung tissues were collected. The tissues were homogenised, serially diluted, and spread on Leeds agar plates. Following overnight incubation at 37°C, the colonies were enumerated, and the organ burden was calculated in terms of colony-forming units (CFU) per gram.

For *in vivo* rifampicin treatment, the animals were infected intranasally with $1 × 10^8$ CFU of the indicated strains, prepared as mentioned above. Following infection, the animals were divided into two groups: treated and untreated. Animals from the treatment group received rifampicin treatment at 5mg/kg of body weight at 2, 14, and 26 hours post-infection. 36 hours post-infection, the mice were sacrificed, and the lung tissues were collected. The tissues were homogenised, serially diluted, and spread on Leeds agar plates. Following overnight incubation at 37°C, the colonies were enumerated, and the organ burden was calculated in terms of colony-forming units (CFU) per gram. The difference in organ burden between the treatment group and the untreated group was further calculated by subtracting the organ burden of the untreated group from that of the treatment group.

For the preparation of inoculum for animal infection studies with the wild-type and Δ*cysE*-Δ*sat*-Δ*05235* strains, the cells were grown in LB broth supplemented with 200 µM cysteine. Overnight-grown cells were freshly inoculated in LB broth supplemented with 200 µM cysteine with 0.1% inoculum and incubated till the mid-log phase. Cells were harvested and washed twice with normal saline and resuspended in normal saline to achieve a final concentration of $5×10^9$ CFU/mL. Each mouse was infected intranasally with 20 µL of this suspension, delivering $1×10^8$ CFU per animal. 36 hours post-infection, the mice were sacrificed, and the lung tissues were collected. The tissues were homogenised, serially diluted, and spread on Leeds agar plates. Following overnight incubation at 37°C, the colonies were enumerated, and the organ burden was calculated in terms of colony-forming units (CFU) per gram.

Supporting tables:

Table S1: Strains used in this study

Sl. No.	Strains	Name used in this study	Description	Reference
1	*Acinetobacter baumannii* ATCC 17978 UN	Wild type/WT	Wild-type (WT) strain, UN (AbaAL44+)	ATCC, USA
2	*Acinetobacter baumannii* ATCC 17978 UN Δ*cysE*	Δ*cysE*	Deletion mutant of *cysE*	This study
3	*Acinetobacter baumannii* ATCC 17978 UN Δ*sat*	Δ*sat*	Deletion mutant of *sat*	This study
4	*Acinetobacter baumannii* ATCC 17978 UN Δ*cysE*-Δ*sat*	Δ*cysE*-Δ*sat*	Deletion mutant of cysE and *sat*	This study
5	*Acinetobacter baumannii* ATCC 17978 UN Δ*cysE*-Δ*sat*::*cysE*	Δ*cysE*-Δ*sat*::*cysE*	Δ*cysE*-Δ*sat* strain complemented with a functional copy of *cysE* in the genome, in the original *cysE* locus.	This study
6	*Acinetobacter baumannii* ATCC 17978 UN Δ*05235*	Δ*05235*	Deletion mutant of l-cystine transporter (KZA74_05235)	This study
7	*Acinetobacter baumannii* ATCC 17978 UN Δ*cysE*-Δ*05235*	Δ*cysE*-Δ*05235*	Deletion mutant of *cysE* and l-cystine transporter (KZA74_05235)	This study

8	*Acinetobacter baumannii* ATCC 17978 UN Δ*cysE*-Δ*sat*-Δ*05235*	Δ*cysE*-Δ*sat*-Δ*05235*	Deletion mutant of *cysE*, *sat* and l-cystine transporter (KZA74_05235)	This study
9	*E. coli* DH5α	*E. coli* DH5α	supE44 hsdR17 recA1 endA1 gyrA96 thi-1, cloning vector	Invitrogen, USA
10	*E. coli* BL21DE3	*E. coli* BL21DE3	hsdS gal (cIts857 ind1 Sam7 nin5 lacUV5-T7 gene 1) , expression vector	Invitrogen, USA

Table S2: Plasmids used in this study

Sl. No.	Plasmid name	Relevant Characteristics	Source/ reference
1	pET28c	Vector for expression of his-tagged recombinant proteins in *E. coli*. kan^r	Novagen
2	pET28c-*cysE*	*cysE* ORF cloned in pET28c	This study
3	pET28c-*sat*	*sat* ORF cloned in pET28c	This study
4	pET28c-*cysK*	*cysK* ORF cloned in pET28c	This study
5	pET28c-*cysM*	*cysM* ORF cloned in pET28c	This study
6	pUC18	pUC18 vector for cloning knock-out constructs. amp^r	Addgene, USA
7	pMDIAI	Plasmid containing an Apramycin resistance cassette between FRT sites (Apr-FRT cassette)	Addgene, USA
8	pUC18-*cysE* k/o	pUC18 harbouring Upstream 355 bp, 3X FLAG, Apr-FRT cassette, Downstream 475 bp of the 99bp region of cysE to be deleted	This study
9	pUC18-*sat* k/o	pUC18 harbouring Upstream 402 bp, 3X FLAG, Apr-FRT cassette, Downstream 313 bp of the 679bp region of SAT to be deleted	This study
10	pUC18-Δ05235 k/o	Upstream 503bp, 3X FLAG, Apr-FRT, Downstream 500bp of the 1238bp of cystine transporter (KZA74_05235) to be deleted	This study
11	pAT02	Plasmid expressing Rec_{AB} recombinase, contains an ampicillin resistance marker	Prof. Bryan Davies, University of Texas, San Antonio, USA
12	pAT03	Plasmid expressing Flp recombinase, contains an ampicillin resistance marker	Prof. Bryan Davies, University of Texas, San Antonio, USA

Table S3: List of primers used in this study

Sl no.	Name	Sequence (5’-3’)	Purpose
1	*cysE*-pET28-FP(NdeI)	GGAATTCCATATGCTTAAACAGCTTAAAGAAGATATACAGGC	Forward primer to clone *cysE* ORF in pET28c
2	*cysE*-pET28-RP(XhoI)	CCGCTCGAGTTACGTTTTTGATGTCTTATTCTGCGCAC	Reverse primer to clone *cysE* ORF in pET28c
3	*sat*-NdeI-FP-pET28c	GGAATTCCATATGACTCAATGGAACATTAATGCGGTTGTACAAGG	Forward primer to clone *sat* ORF in pET28c

4	*sat*-XhoI-RP-pET28c	CCGCTCGAGTTAACCGGCAATATGATTTTTTTGATAAGAC	Reverse primer to clone *sat* ORF in pET28c
5	*cysK*_pET28_FP	GGATTCCATATGTCTACTGATCCACAATTCCCAAC	Forward primer to clone *cysK* ORF in pET28c
6	*cysK*_pET28_RP	CCGCTCGAGTTATTCGTCAAATAAACCTTC	Reverse primer to clone *cysK* ORF in pET28c
7	*cysM*_pET28C_FP	GGATTCCATATGAGTAATACACAACCTGATTTTTAGC	Forward primer to clone *cysM* ORF in pET28c
8	*cysM*_pET28C_RP	CCGCTCGAGTCAGTCCTGTACTGAGAACAGACC	Reverse primer to clone *cysM* ORF in pET28c
9	*cysE*_up355_FP	CCAAGCTTGCTTCTGATGCTGGGCAAATGC	Forward primer to amplify upstream 355 bp of *cysE* (the portion to be deleted)
10	*cysE*_up355_RP	GCGTCGACCTATCATTATGCAGCAGGATCTCGC	Reverse primer to amplify upstream 355 bp of *cysE* (the portion to be deleted)
11	*cysE*_Apr-FRT_RP	CAAAACGGGCGTGTAGGCTGGAGCTGCTTC	Reverse primer to amplify the Apr-FRT cassette for *cysE*
12	*cysE*_dn475_FP	CAGCCTACACGCCCGTTTTGTTTCGTCATTTAGTC	Forward primer to amplify the downstream 475 bp of *cysE* (the portion to be deleted)
13	*cysE*_dn475_RP	TATGACCATGATTACGAATTCCTCGCATTCCTTCTAAAATTGGGTC	Reverse primer to amplify the downstream 475 bp of *cysE* (the portion to be deleted)
14	Amp_*cysE*_FP	GATCGTATGGAATATCATTTACTTCGCGG	Forward primer to amplify the *cysE* knock-out construct
15	N_Amp_*cysE*_RP	CCTAAGGTAACACCGTGGTATAG	Reverse primer to amplify the *cysE* knock-out construct
16	UP_402_*sat*_FP	CGCAAGCTTGGCAGTTTATCACCTGTTG	Forward primer to clone upstream 402 of *sat* (the portion to be deleted)
17	UP_402_*sat*_RP	CGCGTCGACTCATTATTACACTGAAAATTGATTGAAG	Reverse primer to clone upstream 402 of *sat* (the portion to be deleted)
18	*sat*_Apr-FRT_Inf_RP	TGATAAATACGTGTAGGCTGGAGCTGCTTC	Reverse primer to clone Apr-FRT cassette *sat*
19	*sat*_DN313_Inf_FP	CAGCCTACACGTATTTATCAGGCCGTGACCC	Forward primer to clone downstream 313 of *sat* (the portion to be deleted)
20	*sat*_DN313_Inf_RP	TATGACCATGATTACGAATTCGCACGTTCTTTGTTTATTTATCTCC	Reverse primer to clone downstream 313 bp of *sat* (the portion to be deleted)
21	Amp_*sat*_KO_FP	AAGCTGCCGAAACCGCATTTAATG	Forward primer to amplify *sat* knock-out construct
22	Amp_*sat*_RP	CCAATAATTGACCCTCGACCAATGGTAATACGGC	Reverse primer to amplify *sat* knock-out construct
23	05235_F1_FP	CCCAAGCTTTCCTATCGGCATGCAATTGAG	Forward primer to amplify the upstream 503 bp of the cystine transporter (the portion to be deleted)
24	05235_F1_RP	GCGTCGACTTATTACATTCCCAAACTCCAAATTATTG	Reverse primer to amplify the upstream 503 bp of the cystine transporter (the portion to be deleted)

25	05235_F3_RP	AATTGAAATAGTGTAGGCTGGAGCTGCTTC	Reverse primer to clone the Apr-FRT cassette for the cystine transporter
26	05235_F4_FP	CAGCCTACACTATTTCAATTGAACCACTCATTGA	Forward primer to amplify the downstream 500 bp of the cystine transporter (the portion to be deleted)
27	05235_F4_RP	TATGACCATGATTACGAATTCCTCAACCGTTGGAGTAAACCAA	Reverse primer to amplify the downstream 500 bp of the cystine transporter(the portion to be deleted)
28	Amp 05235 k/o FP	CAAAAGTCATTTTATATTCTCATTCAG	Forward primer to amplify the cystine transporter knock-out construct
29	Amp 05235 k/o RP	GTTGAGTTAATTCAATTTCATCTTCG	Reverse primer to amplify the cystine transporter knock-out construct
30	FLAG_FP(SalI)	CGCGTCGACGACTACAAGGACCACGAC	Forward primer to amplify the FLAG-tag
31	KpnI-3X FLAG-RP	CCGGTACCAAAAAAAAGGCCGGGGGAGC	Reverse primer to amplify the FLAG tag
32	KpnI_APR-FRT_Inf_FP	CTCCCCCGGCCTTTTTTTTGGTACCCTGCAGTTCGAAGTTCCTATTCTC	Forward primer to amplify the Apr-FRT cassette
33	RT_*sat*_FP	TTATCAGGCCGTGACCCTTG	Forward RT primer for *sat* gene
34	RT_*sat*_RP	GGCACCTGCATAAATCACGAC	Reverse RT primer for *sat* gene
35	RT_*sat* DN_FP	CCGGGGCTTTCAGTACGTTT	Forward RT primer for the downstream gene of *sat*
36	RT_*sat* DN_RP	CACTGCCAAAGCATCGTCTG	Reverse RT primer for the downstream gene of *sat*
37	RT_*sat* UP_FP	TTATGCCGTAGTGGCAACCG	Forward RT primer for the upstream gene of *sat*
38	RT_*sat* UP_RP	ACGCTGTTGCTGTTCATTTAGA	Reverse RT primer for the upstream gene of *sat*
39	RT_*cysE* DN_FP	GCAGCGGCTAAATATGCTTCC	Forward RT primer for the downstream gene of *cysE*
40	RT_*cysE* DN_RP	GCTTATCTGAAGGTGGCGGT	Reverse RT primer for the downstream gene of *cysE*
41	RT_*cysE* UP_FP	ACGACGCAAGCGTAAAGGTA	Forward RT primer for the upstream gene of *cysE*
42	RT_*cysE* UP_RP	CCGTTAGTGACTCAGCAGCA	Reverse RT primer for the upstream gene of cysE
43	*cysE*_Comp_I1_RP	GCGTCGACGACATGCAAGGGTAAGC	To amplify upstream 298bp+ *cysE* ORF+41bp downstream
44	*cysE*_Comp_I2_FP	GCGTCGACCTGCAGTTCGAAGTTCCTATTC	To amplify Apr-FRT cassette
45	*cysE*_Comp_I2_RP	CGGGATCCGTGTAGGCTGGAGCTGCTTC	To amplify Apr-FRT cassette
46	*cysE*_Comp_I3_FP	CGGGATCCGGTTTAGGTTTTCCTAGACTTG	To amplify downstream 459 bp
47	*cysE*_Comp_I3_RP	CGGAATTCAGTGACGCGCTTTACCTTCAAC	To amplify downstream 459 bp
48	Amp_*cysE* comp_FP	CGTTTATTTGGTCGTATACAATTAGATCG	To amplify *cysE* knock-in construct
49	Amp_*cysE* comp_RP	CATGGTACACGCTGTTAAACTTGAGGTCG	To amplify *cysE* knock-in construct
50	RT_*cysW*_FP	TGGAAGCGCAAGGAACTGAA	Forward RT primer for *cysW*
51	RT_*cysW*_RP	CAAACTCACCCATTGCACGG	Reverse RT primer for *cysW*
52	RT_*cysT*_FP	TTTAGCATTTGCTCGCGGTG	Forward RT primer for *cysT*

53	RT_*cysT*_RP	TGCTGCAATAGTCGTTGCAC	Reverse RT primer for *cysT*
54	RT_*cysA*_FP	TTACCGACCACAGTTCAGGC	Forward RT primer for *cysA*
55	RT_*cysA*_RP	TCCGCCACTACTTTTCCAGC	Reverse RT primer for *cysA*
56	RT_*cysB*_FP	TACGTTGAACTCGGCATGGG	Forward RT primer for *cbl*
57	RT_*cysB*_RP	AAATGGCCTTTGCGAACAGC	Reverse RT primer for *cbl*
58	KZA74_RS00900_RT_FP	AGCAGTAAATGCGTTTGGCG	Forward RT primer for *sfnG*
59	KZA74_RS00900_RT_RP	GCAATTTGCTGAGGTGTACCA	Reverse RT primer for *sfnG*
60	KZA74_RS10980_RT_FP	TGGTGCAGGTGGTCTAGGAG	Forward RT primer for methionine ABC transporter permease
61	KZA74_RS10980_RT_RP	TGGGCTAATGCATCACCAGTC	Reverse RT primer for methionine ABC transporter permease
62	KZA74_RS18400_RT_FP	GGCGTTGTAGAAGCTGATACTC	Forward RT primer for *grpE*
63	KZA74_RS18400_RT_RP	ACCATAGCAGGGCGCAATAA	Reverse RT primer for *grpE*
64	KZA74_RS09875_RT_FP	AACCCTAGTCGCAGGTCTTG	Forward RT primer for sulfite exporter
65	KZA74_RS09875_RT_RP	AACGTTCTGGCATACGTGAG	Reverse RT primer for sulfite exporter
66	RT_*gigC*_FP	GGCGGGTAAGTTGCCAAAAT	Forward RT primer for *gigC*
67	RT_*gigC*_RP	ATAGTGGCTGGCGGTTTAGG	Reverse RT primer for *gigC*
68	RT_*cysB*_FP	TACGTTGAACTCGGCATGGG	Forward RT primer for *cbl*
69	RT_*cysB*_RP	AAATGGCCTTTGCGAACAGC	Reverse RT primer for *cbl*
70	RT_05235_FP	ATTGAAGCGCAAACTCGTCG	Forward RT primer for cystine transporter
71	RT_05235_RP	TAGTTGGCGCAACCATGACT	Reverse RT primer for cystine transporter
72	HPF_RT_FP	GTTGCAGTGCACCATAAAGCA	Forward RT primer for hibernation promoting factor
73	HPF_RT_RP	TCTGGTTGACCACCTCTCGG	Reverse RT primer for hibernation promoting factor

Table S4: Genes showing differential transcript abundance in Δ*cysE*-Δ*sat* compared to wild-type strain (P value<0.05 and log_2 fold change>1.5)

Category/ function	Upregulated genes			Downregulated genes		
	Locus tag.	Annotation	Fold change(log2)	Locus tag.	Annotation	Fold change(log2)
Methionine biosynthesis and uptake	KZA74_10975	MetQ/NlpA family ABC transporter substrate-binding protein	8.282796058			
	KZA74_10990	Methionine ABC transporter permease	8.140460301			
	KZA74_10985	Methionine ABC transporter ATP-binding protein	7.71904044			
	KZA74_10980	MetQ/NlpA family ABC transporter substrate-binding protein	7.415423416			
	KZA74_06655	MetQ/NlpA family ABC transporter substrate-binding protein	1.681695372			
	KZA74_15015	methionine synthase	4.716980498			
	KZA74_13245	MetQ/NlpA family ABC transporter substrate-binding protein	3.169829604			
	KZA74_14065	homocysteine S-methyltransferase family protein	4.390124441			
Sulfonate and sulfur metabolism and uptake	KZA74_00900	dimethylsulfone monooxygenase SfnG	9.322740186	KZA74_09885	sulfite exporter TauE/SafE family protein	-1.926496828
	KZA74_18300	aliphatic sulfonate ABC transporter permease	7.951603486	KZA74_09515	thiolase family protein	-1.690109328
	KZA74_18295	FMNH2-dependent alkanesulfonate monooxygenase	7.915871682			
	KZA74_10965	SfnB family sulfur acquisition oxidoreductase	7.681650029			
	KZA74_18285	sulfonate ABC transporter substrate-binding protein	7.207624239			
	KZA74_10960	SfnB family sulfur acquisition oxidoreductase	7.107744985			
	KZA74_18290	Sulfonate ABC transporter substrate-binding protein	7.284781718			
	KZA74_01345	Bifunctional SulP family inorganic anion transporter/carbonic anhydrase	2.913606154			
	KZA74_11400	Cysteine ABC transporter substrate-binding protein	5.133560751			
	KZA74_11375	Family 2A encapsulin nanocompartment cargo protein cysteine desulfurase	2.468133174			
	KZA74_13785	Arylsulfatase	7.645821705			

	KZA74_01370	O-acetylhomoserine aminocarboxypropyltransferase/cysteine synthase family protein	3.411183558			
	KZA74_01465	Sulfate ABC transporter substrate-binding protein	5.602461027			
	KZA74_04905	Sulfate ABC transporter permease subunit CysW	4.454748552			
	KZA74_04900	Sulfate/molybdate ABC transporter ATP-binding protein	4.347756145			
	KZA74_04920	Sulfate ABC transporter substrate-binding protein	4.271811244			
	KZA74_04910	Sulfate ABC transporter permease subunit CysT	4.12039499			
	KZA74_16470	SfnB family sulfur acquisition oxidoreductase	9.518044588			
	KZA74_03270	Nitrite/sulfite reductase	1.518240745			
Transcriptional regulators	KZA74_09590	GntR family transcriptional regulator	7.636439939	KZA74_09455	AraC family transcriptional regulator	-1.523367054
	KZA74_04895	CysB family HTH-type transcriptional regulator (Cbl)	3.71415123	KZA74_12870	transcriptional regulator	-1.542281931
	KZA74_06980	LysR family transcriptional regulator	1.662298852	KZA74_12060	TetR/AcrR family transcriptional regulator	-1.610001131
	KZA74_00895	Lrp/AsnC family transcriptional regulator	1.552026676	KZA74_06505	TetR/AcrR family transcriptional regulator	-2.683449724
	KZA74_11390	LysR family transcriptional regulator	4.360364935	KZA74_04340	TetR/AcrR family transcriptional regulator	-3.035943639
	KZA74_10730	putA	2.087831665	KZA74_07185	TetR/AcrR family transcriptional regulator	-1.771100917
	KZA74_15345	MarR family winged helix-turn-helix transcriptional regulator	2.070077077	KZA74_05345	Lrp/AsnC family transcriptional regulator	-2.413627275
	KZA74_14040	Transcriptional regulator BetI	2.481134485			
Urea metabolism				KZA74_13615	urease accessory protein UreD	-2.129624572
				KZA74_00665	2-oxo-4-hydroxy-4-carboxy-5-ureidoimidazoline decarboxylase	-2.900130116
				KZA74_12045	urea amidolyase associated protein UAAP1	-2.904974472
				KZA74_13605	urease subunit beta	-1.844203652

				KZA74_13610	urease subunit gamma	-1.866389503
Transporters and associated proteins	KZA74_09575	ABC transporter substrate-binding protein	7.39609507	KZA74_13260	Na+/H+ antiporter NhaC family protein	-2.898319257
	KZA74_09570	ABC transporter permease	6.405017369	KZA74_06060	ABC transporter substrate-binding protein	-1.637086652
	KZA74_09565	ABC transporter ATP-binding protein	6.403657376	KZA74_00930	cation acetate symporter	-1.657486229
	KZA74_11415	amino acid ABC transporter permease	4.962741239	KZA74_08945	MFS transporter	-1.667002006
	KZA74_11420	amino acid ABC transporter permease	4.850966495	KZA74_08555	spinster family MFS transporter	-1.672026698
	KZA74_10165	ABC transporter substrate-binding protein	2.769464635	KZA74_07800	ABC transporter substrate-binding protein	-1.920830621
	KZA74_10170	ABC transporter substrate-binding protein	2.612734577	KZA74_03780	ammonium transporter	-2.247719658
	KZA74_10145	ABC transporter ATP-binding protein	2.435363251	KZA74_09510	short-chain fatty acid transporter	-2.612300195
	KZA74_06620	MFS transporter	1.567664799			
	KZA74_11405	transporter substrate-binding domain-containing protein	5.064694733			
	KZA74_05225	YbfB/YjiJ family MFS transporter	4.257788529			
	KZA74_10150	ABC transporter permease	2.36495816			
	KZA74_03755	DcaP family trimeric outer membrane transporter	2.22618181			
	KZA74_10155	ABC transporter permease	2.160613334			
	KZA74_00200	MFS transporter	2.053821102			
	KZA74_09595	chromate efflux transporter	2.00517416			
	KZA74_11410	amino acid ABC transporter ATP-binding protein	5.11521883			
	KZA74_01015	amino acid permease	3.327568513			
	KZA74_10190	MotA/TolQ/ExbB proton channel family protein	4.82821515			
	KZA74_01225	aquaporin Z	1.986193475			
	KZA74_05780	TonB-dependent receptor plug domain-containing protein	7.402725282			
	KZA74_00945	porin Omp33-36	1.823372375			
	KZA74_10180	TonB-dependent receptor	5.299684056			
	KZA74_17410	OprD family outer membrane porin	3.035684604			
	KZA74_14070	basic amino acid/polyamine antiporter	4.163290975			
	KZA74_17570	TonB-dependent copper receptor	2.129195581			

	KZA74_18305	ABC transporter ATP-binding protein	7.459156434			
	KZA74_17610	MFS transporter	2.629963879			
Histidine metabolism	KZA74_00375	histidine utilization repressor	1.911874987			
	KZA74_00380	HutD family protein	1.870509675			
Chaperones				KZA74_04215	co-chaperone GroES	-1.687678315
				KZA74_10845	fimbrial biogenesis chaperone	-1.90266589
				KZA74_12565	ATP-dependent chaperone ClpB	-2.68871187
				KZA74_18410	nucleotide exchange factor GrpE	-2.040752589
csu genes (biofilm related)				KZA74_06530	Csu fimbrial usher CsuD	-2.804265759
				KZA74_06525	Csu fimbrial biogenesis chaperone CsuC	-3.564355614
				KZA74_06510	Csu fimbrial major subunit CsuAB	-4.847288501
				KZA74_06535	Csu fimbrial tip adhesin CsuE	-1.737790053
				KZA74_10840	fimbrial protein	-1.800997953
Reductases and oxidoreductases	KZA74_10970	LLM class flavin-dependent oxidoreductase	8.383326304	KZA74_09380	SDR family oxidoreductase	-1.817158202
	KZA74_00905	FMN reductase	9.714296677	KZA74_13215	NAD(P)/FAD-dependent oxidoreductase	-1.584002047
	KZA74_09585	fumarate reductase/succinate dehydrogenase flavoprotein subunit	7.869140381			
	KZA74_08235	nitroreductase family protein	1.609654289			
	KZA74_06105	ferredoxin--NADP reductase	1.56565653			
	KZA74_00205	2,5-didehydrogluconate reductase DkgB	1.867958192			
	KZA74_15010	Flavin reductsase	3.452127901			
	KZA74_09630	Bfunctional protein tyrosine phosphatase family protein/NAD(P)/FAD-dependent oxidoreductase	2.595813197			
Peptidase, kinase, transaminase, and	KZA74_05785	M1 family metallopeptidase	5.101223651	KZA74_02275	M3 family metallopeptidase	-1.704345423
	KZA74_01850	S8 family serine peptidase	3.250385891	KZA74_07130	aminopeptidase N	-1.802184235

dehydrogenase	KZA74_10160	M3 family metallopeptidase	2.063487328	KZA74_11560	isovaleryl-CoA dehydrogenase	-1.722387209
	KZA74_03575	M13 family metallopeptidase	1.9580385	KZA74_09675	thiamine pyrophosphate-dependent dehydrogenase E1 component subunit alpha	-2.174091933
	KZA74_01025	4-aminobutyrate--2-oxoglutarate transaminase	5.304765553			
	KZA74_14045	betaine-aldehyde dehydrogenase	1.976410342			
	KZA74_14050	choline dehydrogenase	1.839100119			
	KZA74_01030	NAD-dependent succinate-semialdehyde dehydrogenase	2.144825739			
Energy transduction	KZA74_09580	4Fe-4S dicluster domain-containing protein	7.971195463			
	KZA74_10185	energy transducer TonB	4.718420287			
	KZA74_10195	ExbD/TolR family protein	4.415334897			
	KZA74_10200	ExbD/TolR family protein	4.408363757			
**Vitamin biosynthesis**	KZA74_14650	adenosylmethionine--8-amino-7-oxononanoate transaminase	1.996023392			
	KZA74_14645	adenosylmethionine--8-amino-7-oxononanoate transaminase	1.810211672			
DUF domain-containing proteins and hypothetical proteins	KZA74_00410	DUF885 domain-containing protein	3.593639428	KZA74_12290	DUF4142 domain-containing protein	-1.629870945
	KZA74_03275	DUF934 domain-containing protein	2.265555945	KZA74_07710	DUF1833 family protein	-2.030166059
	KZA74_00265	DUF1328 domain-containing protein	1.823087061	KZA74_06430	DUF2171 domain-containing protein	-2.406316257
	KZA74_07690	DUF4236 domain-containing protein	1.570032811	KZA74_04785	hypothetical protein	-1.551830475
	KZA74_15020	DUF1852 domain-containing protein	4.318898248	KZA74_07485	hypothetical protein	-1.66864342
	KZA74_13795	hypothetical protein	4.444855601	KZA74_09735	hypothetical protein	-1.800483806
	KZA74_10175	hypothetical protein	4.3517037	KZA74_00435	hypothetical protein	-1.884955123
	KZA74_06465	hypothetical protein	3.385004371	KZA74_02405	hypothetical protein	-2.112529844
	KZA74_01845	hypothetical protein	3.100745822	KZA74_06955	hypothetical protein	-2.130819443
	KZA74_07685	YdcA family protein	2.843596485	KZA74_04505	hypothetical protein	-2.194647808

	KZA74_08165	hypothetical protein	1.555286381	KZA74_02705	hypothetical protein	-2.555294536
				KZA74_06635	hypothetical protein	-3.174141859
				KZA74_12325	hypothetical protein	-3.187800587
				KZA74_12395	hypothetical protein	-3.976638604
				KZA74_12285	hypothetical protein	-1.582886007
				KZA74_13300	hypothetical protein	-1.731496738
				KZA74_13165	hypothetical protein	-2.160783298

Supporting figures:

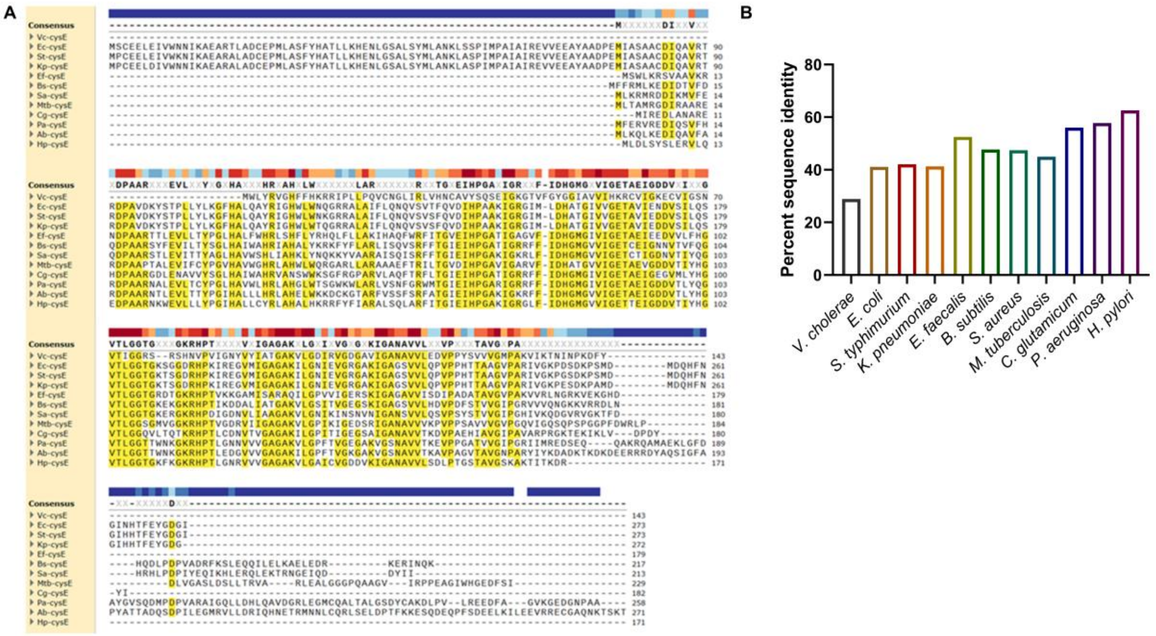

Figure S1: Similarity of *A. baumannii* serine O-acetyltransferase (CysE) protein with serine acetyltransferases from other Gram-negative and Gram-positive bacterial species

(A) Multiple sequence alignment and (B) percent similarity of *A. baumannii* serine O-acetyltransferase (CysE) protein with serine acetyltransferases of other Gram-negative and Gram-positive bacteria (Vc-*Vibrio cholerae*, Ec-*Escherichia coli*, St-*Salmonella typhimurium*, Kp-*Klebsiella pneumoniae*, Ef-*Enterococcus faecalis*, Bs-*Bacillus subtilis*, Sa-*Staphylococcus aureus*, Mtb-*Mycobacterium tuberculosis*, Cg-*Corynebacterium glutamicum*, Pa-*Pseudomonas aeruginosa*, Ab-*Acinetobacter baumannii* and Hp-*Helicobacter pylori*).

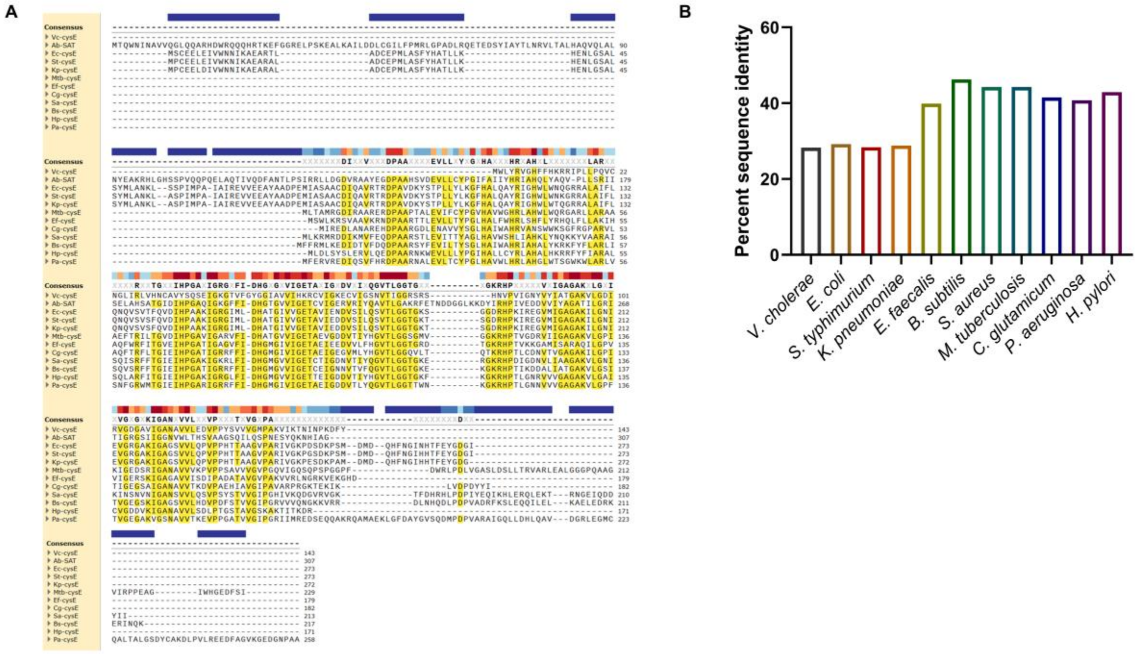

Figure S2: Similarity of *A. baumannii* serine acetyltransferase (SAT) protein with serine acetyltransferases from other Gram-negative and Gram-positive bacterial species

(A) Multiple sequence alignment and (B) percent similarity of *A. baumannii* serine acetyltransferase (SAT) protein with serine acetyltransferases of other Gram-negative and Gram-positive bacteria (Vc-*Vibrio cholerae*, Ec-*Escherichia coli*, St-*Salmonella typhimurium*, Kp-*Klebsiella pneumoniae*, Ef-*Enterococcus faecalis*, Bs-*Bacillus subtilis*, Sa-*Staphylococcus aureus*, Mtb-*Mycobacterium tuberculosis*, Cg-*Corynebacterium glutamicum*, Pa-*Pseudomonas aeruginosa*, Ab-*Acinetobacter baumannii* and Hp-*Helicobacter pylori*).

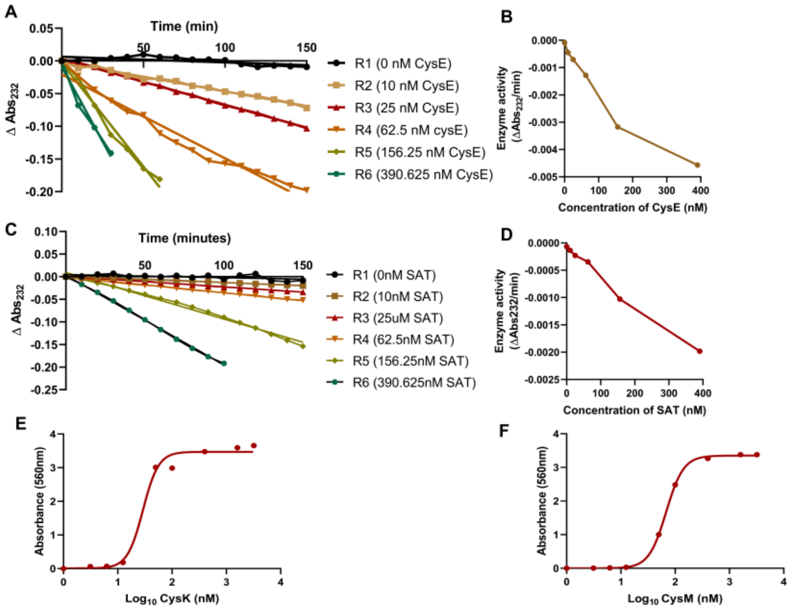

Figure S3: *Acinetobacter baumannii* serine acetyltransferase genes *cysE* and *sat,* and cysteine synthase genes *cysK and cysM,* encode functional enzymes (A) Graph depicting the degradation of acetyl-CoA with time, as indicated by the decrease in absorbance at 232 nm, following incubation with varying concentrations of purified CysE. (B)

Slope representing enzyme activity (ΔAbs_{232}/minute) at different concentrations of CysE. (C) Graph depicting the degradation of acetyl-CoA with time, as indicated by the decrease in absorbance at 232 nm, following incubation with varying concentrations of purified SAT. (D) Slope representing enzyme activity (ΔAbs_{232}/minute) at different concentrations of SAT. (E) Graph depicting absorbance at 560 nm of Ruhemann's purple formed upon reaction of ninhydrin with cysteine produced by varying concentrations of CysK. (F) Graph depicting absorbance at 560 nm of Ruhemann's purple formed upon reaction of ninhydrin with cysteine produced by varying concentrations of CysM. Each point represents the average of two values.

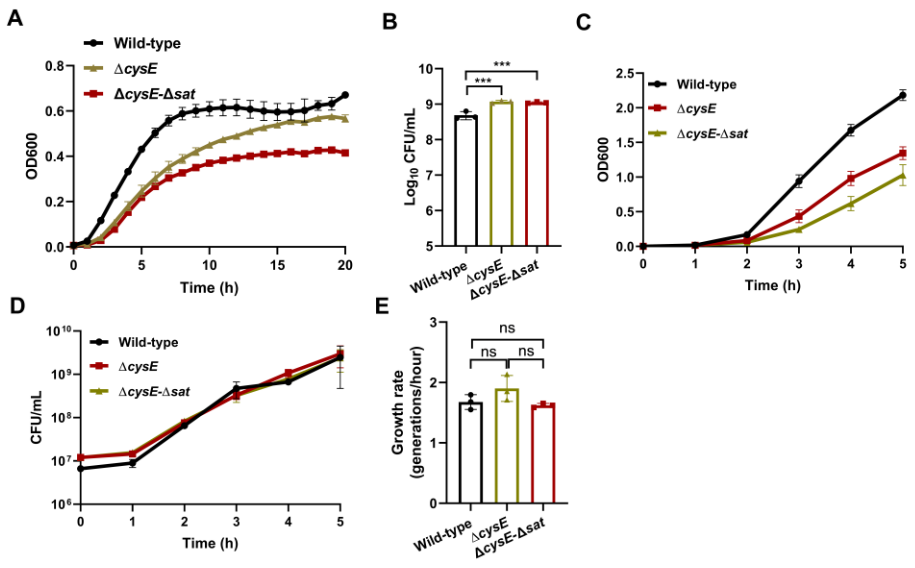

Fig. S4 Cysteine biosynthesis mutants do not show any growth defect in complex medium (A) Growth profile analysis and (B) endpoint CFU/mL of wild type, Δ*cysE*, and Δ*cysE*-Δ*sat* in LB broth, indicating that variations in OD600 do not correspond to similar differences in viable cell counts. The assay was performed in a 96-well plate at 37°C with continuous shaking, and the optical density (OD600) value was measured every hour using a Synergy H1 plate reader (Agilent). Samples were collected at the endpoint, then diluted and spread on LB agar plates to determine the viable cell count. Each point represents the mean of three values with SD shown as error bars. Statistical significance was measured using one-way ANOVA with multiple comparisons, where *** represents p-value <0.001. Growth profile analysis of wild-type, Δ*cysE* and Δ*cysE*-Δ*sat* depicting OD600 (C) and CFU/mL (D). Cells were grown in an incubator at 37°C with shaking at 180 rpm. Samples were collected at the indicated time points, and OD600 and CFU were measured. Each point represents the mean of three values with SD shown as error bars. (E) Growth rate of wild type, Δ*cysE* and Δ*cysE*-Δ*sat* calculated from CFU value of logarithmic phase cells between 2 and 4 hours. The data represent values from three biological replicates. Statistical significance was measured using one-way ANOVA with multiple comparisons, where ns represents non-significant.

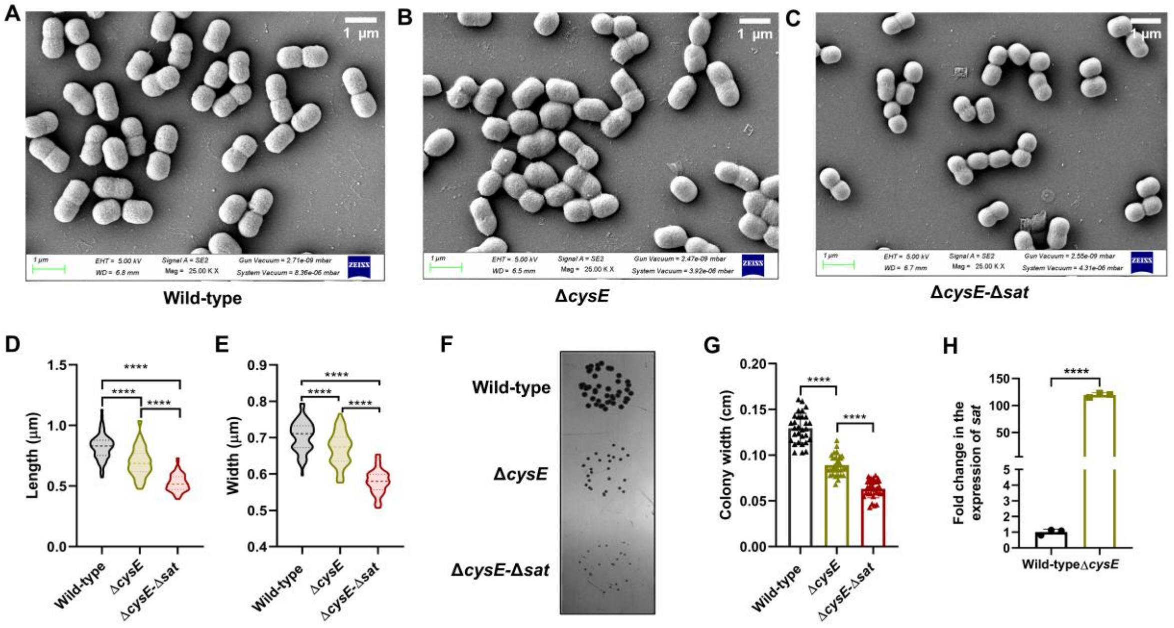

Figure S5. Disruption of cysteine biosynthesis affects cellular morphology and colony size in *A. baumannii.*

Scanning electron micrographs of (A) wild type, (b) Δ*cysE*, and (C) Δ*cysE*-Δ*sat* cells at mid-log phase visualised at 25,000× magnification. Cell length (D) and width (E) were quantified using ImageJ with reference to the scale and plotted using GraphPad Prism (n=70 cells). Statistical significance was measured using one-way ANOVA with multiple comparisons, where **** represents p-value <0.0001. (F) Image of colonies formed by wild-type, Δ*cysE* and Δ*cysE*-Δ*sat* strains on an LB agar plate. Cells were grown in LB broth till mid-log phase, diluted, and spotted onto LB agar plates and incubated for 23 hours. (G) Bar graph representing the distribution of colony widths measured using ImageJ and plotted using GraphPad Prism (n=30 colonies). Statistical significance was measured using one-way ANOVA with multiple comparisons, where **** represents p-value <0.0001. (H) RT-PCR data representing changes in the expression of *sat* in the Δ*cysE* strain compared to the wild type when grown in LB broth. The data represent three values, with the SD shown as error bars. Statistical significance was measured using a t-test, where **** represents p-value <0.0001.

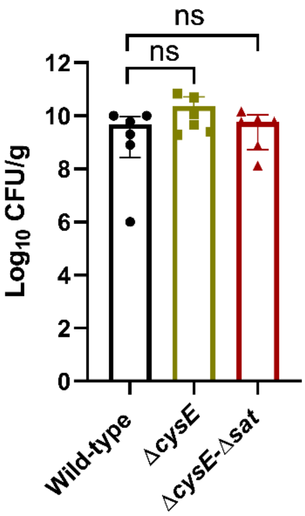

Figure S6: Cysteine biosynthesis is not essential for the virulence of *A. baumannii* in a murine pneumonia infection model.

Enumeration of bacterial burden in mice lungs infected with wild-type, Δ*cysE* and Δ*cysE*-Δ*sat* strains at 36 hours post-infection. n=6. Statistical significance was measured using one-way ANOVA with multiple comparisons. ns represents non-significant.

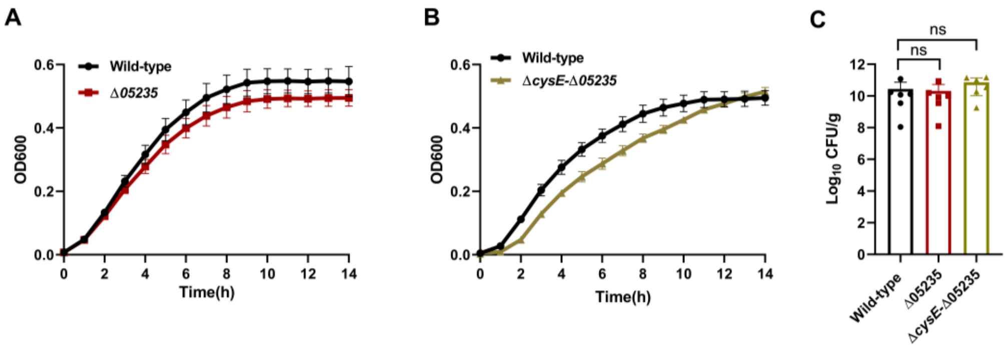

Figure S7: **Cystine transporter KZA74_05235 is not essential for growth and virulence in the presence of a functional biosynthetic enzyme**

Growth profile analysis of the wild-type and Δ05235 (A) and wild-type and Δ*cysE*-Δ05235 (B) strains in LB broth. (C)Enumeration of bacterial burden in mice lungs infected with the wild-type, Δ*05235* and Δ*cysE*-Δ*05235* strains at 36 hours post-infection. n=6. Statistical significance was measured using one-way ANOVA with multiple comparisons. ns represents non-significant.

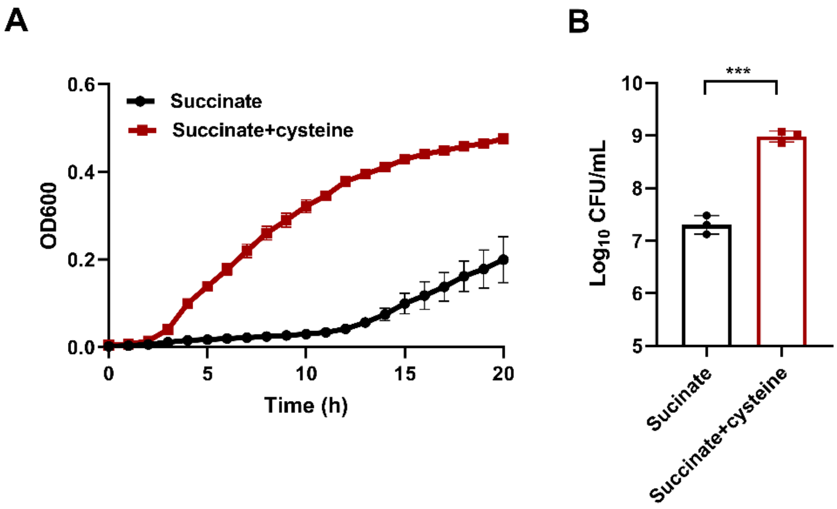

Figure S8: *A. baumannii* can uptake cysteine in the absence of the cystine transporter KZA74_05235.

Growth profile analysis of Δ*cysE*-Δ*05235* in succinate and succinate + 100 μM cysteine, depicting OD600 values over time (A) and endpoint CFU/mL (B). The growth profile analysis was done in a 96-well plate with continuous shaking in a Synergy plate reader (Agilent). Aliquots from the culture at endpoint were serially diluted, and CFU were enumerated. Each point represents the mean of three values with SD shown as error bars. Statistical significance was measured using a t-test, where *** represents p-value <0.001.

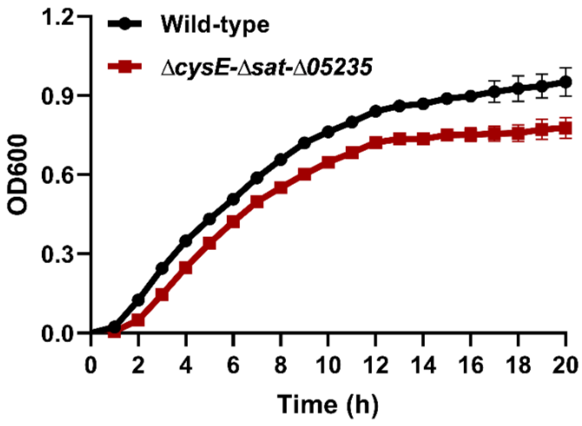

Figure S9: The Δ*cysE*-Δ*sat*-Δ*05235* strain can grow in LB broth supplemented with exogenous cysteine.

Growth profile analysis of wild type and Δ*cysE*-Δ*sat*-Δ*05235* in LB broth supplemented with 200 μM cysteine. Each point represents the mean of three values with SD shown as error bars. The Δ*cysE*-Δ*sat*-Δ*05235* strain can grow in the presence of high concentrations of exogenous cysteine, possibly due to transport through low-affinity or non-specific transporters.

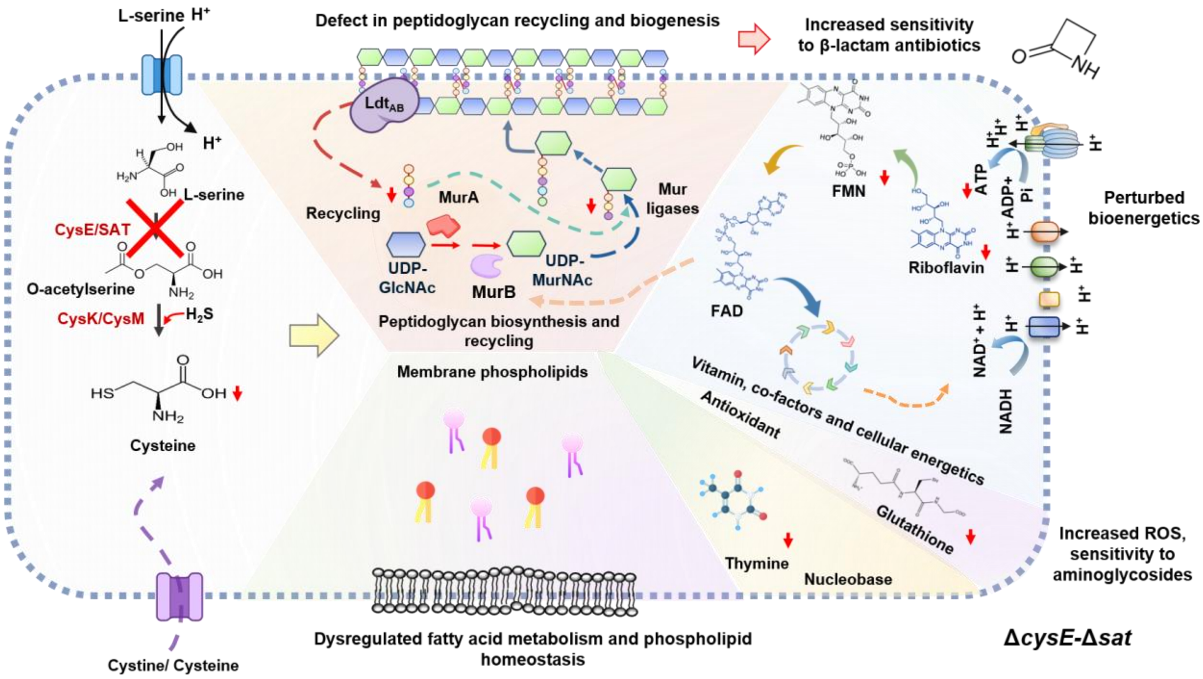

Figure S10: Schematic representation of metabolic perturbation in the Δ*cysE*-Δ*sat* strain. Disruption of cysteine biosynthesis results in a substantial depletion of the intracellular cysteine pool and leads to widespread metabolic perturbations. The Δ*cysE*-Δ*sat* double mutant shows reduced levels of key metabolites, including peptidoglycan precursors, vitamins, antioxidants, and cofactors. Several enzymes involved in peptidoglycan recycling and biogenesis, as well as cofactor biosynthesis, contain cysteine residues in their active sites; thus, cysteine depletion likely compromises the abundance of these enzymes, further contributing to reduced product levels. The depletion of glutathione and cysteine leads to elevated ROS and heightened susceptibility to antibiotics that generate ROS in addition to acting on their canonical targets. Furthermore, defects in peptidoglycan recycling and biosynthesis deplete the intracellular pool of cell wall precursors, resulting in increased sensitivity to β-lactam antibiotics.

References

1. Sett, A., Maiti, P. K., Garg, K., Hussain, A., Saini, S., Pandey, S., and Pathania, R. (2024) 'GGFGGQ' repeats in Hfq of Acinetobacter baumannii are essential for nutrient utilization and virulence. *Journal of Biological Chemistry*. 10.1016/j.jbc.2024.107895
2. Verma, D., Gupta, S., Saxena, R., Kaur, P., Rachana, R. R., Srivastava, S., and Gupta, V. (2020) Allosteric inhibition and kinetic characterization of Klebsiella pneumoniae CysE: An emerging drug target. *Int J Biol Macromol*. 151, 1240–1249
3. Mino, K., Yamanoue, T., Sakiyama, T., Eisaki, N., Matsuyama, A., and Nakanishi, K. (1999) Purification and characterization of serine acetyltransferase from Escherichia coli partially truncated at the C-terminal region. Biosci. Biotechnol. Biochem. 63, 168–179
4. Tucker, A. T., Nowicki, E. M., Boll, J. M., Knauf, G. A., Burdis, N. C., Stephen Trent, M., and Davies, B. W. (2014) Defining gene-phenotype relationships in acinetobacter baumannii through one-step chromosomal gene inactivation. *mBio*. 5, 1–9

5. Bhowmik, S., Pathak, A., Pandey, S., Devnath, K., Sett, A., Jyoti, N., Bhando, T., Akhter, J., Chugh, S., Singh, R., Sharma, T. K., and Pathania, R. (2025) *Acinetobacter baumannii* represses type VI secretion system through a manganese-dependent small RNA-mediated regulation. *mBio*. 10.1128/mbio.03025-24
6. Coppens, L., and Lavigne, R. (2020) SAPPHIRE: a neural network based classifier for σ70 promoter prediction in Pseudomonas. *BMC Bioinformatics*. 21, 415
7. Andrews, S. (2010) FastQC: a quality control tool for high throughput sequence data.
8. Chen, S., Zhou, Y., Chen, Y., and Gu, J. (2018) fastp: an ultra-fast all-in-one FASTQ preprocessor. *Bioinformatics*. 34, i884–i890
9. Liao, Y., Smyth, G. K., and Shi, W. (2014) featureCounts: an efficient general purpose program for assigning sequence reads to genomic features. *Bioinformatics*. 30, 923–930
10. Love, M. I., Huber, W., and Anders, S. (2014) Moderated estimation of fold change and dispersion for RNA-seq data with DESeq2. *Genome Biol*. 15, 1–21
